# A primary nasopharyngeal three-dimensional air-liquid interface cell culture model of the pseudostratified epithelium reveals differential donor- and cell type-specific susceptibility to Epstein-Barr virus infection

**DOI:** 10.1371/journal.ppat.1009041

**Published:** 2021-04-29

**Authors:** Phillip Ziegler, Yarong Tian, Yulong Bai, Sanna Abrahamsson, Alan Bäckerholm, Alex S. Reznik, Anthony Green, John A. Moore, Stella E. Lee, Michael M. Myerburg, Hyun Jung Park, Ka-Wei Tang, Kathy Ho Yen Shair

**Affiliations:** 1 Cancer Virology Program, University of Pittsburgh Medical Center (UPMC), University of Pittsburgh, Pittsburgh, Pennsylvania, United States of America; 2 Wallenberg Centre for Molecular and Translational Medicine, Sahlgrenska Center for Cancer Research, Department of Infectious Diseases, Institute of Biomedicine, Sahlgrenska Academy, University of Gothenburg, Gothenburg, Sweden; 3 Department of Human Genetics, University of Pittsburgh, Pittsburgh, Pennsylvania, United States of America; 4 University of Pittsburgh Research Histology Services, University of Pittsburgh, Pittsburgh, Pennsylvania, United States of America; 5 UPMC Department of Otolaryngology, University of Pittsburgh, Pittsburgh, Pennsylvania, United States of America; 6 Division of Pulmonary, Allergy, and Critical Care Medicine, University of Pittsburgh, Pittsburgh, Pennsylvania, United States of America; 7 Region Västra Götaland, Sahlgrenska University Hospital, Department of Clinical Microbiology, Gothenburg, Sweden; 8 Department of Microbiology and Molecular Genetics, University of Pittsburgh, Pittsburgh, Pennsylvania, United States of America; Pennsylvania State University College of Medicine, UNITED STATES

## Abstract

Epstein-Barr virus (EBV) is a ubiquitous γ-herpesvirus with latent and lytic cycles. EBV replicates in the stratified epithelium but the nasopharynx is also composed of pseudostratified epithelium with distinct cell types. Latent infection is associated with nasopharyngeal carcinoma (NPC). Here, we show with nasopharyngeal conditionally reprogrammed cells cultured at the air-liquid interface that pseudostratified epithelial cells are susceptible to EBV infection. Donors varied in susceptibility to *de novo* EBV infection, but susceptible cultures also displayed differences with respect to pathogenesis. The cultures from one donor yielded lytic infection but cells from two other donors were positive for EBV-encoded EBERs and negative for other lytic infection markers. All cultures stained positive for the pseudostratified markers CK7, MUC5AC, α-tubulin in cilia, and the EBV epithelial cell receptor Ephrin receptor A2. To define EBV transcriptional programs by cell type and to elucidate latent/lytic infection-differential changes, we performed single cell RNA-sequencing on one EBV-infected culture that resulted in alignment with many EBV transcripts. EBV transcripts represented a small portion of the total transcriptome (~0.17%). All cell types in the pseudostratified epithelium had detectable EBV transcripts with suprabasal cells showing the highest number of reads aligning to many EBV genes. Several restriction factors (*IRF1*, *MX1*, *STAT1*, *C18orf25*) known to limit lytic infection were expressed at lower levels in the lytic subcluster. A third of the differentially-expressed genes in NPC tumors compared to an uninfected pseudostratified ALI culture overlapped with the differentially-expressed genes in the latent subcluster. A third of these commonly perturbed genes were specific to EBV infection and changed in the same direction. Collectively, these findings suggest that the pseudostratified epithelium could harbor EBV infection and that the pseudostratified infection model mirrors many of the transcriptional changes imposed by EBV infection in NPC.

## Author summary

It has been known for over 50 years that EBV infection is associated with NPC. Despite many advances from studies in 2-dimensional cell culture, many aspects of EBV molecular pathogenesis in the nasopharynx remain undefined because the cell types and the biology of the nasopharyngeal epithelium can only be faithfully captured in 3-dimensional cell culture. In the stratified epithelium, cellular differentiation triggers lytic infection but it is not clear to what degree the pseudostratified epithelium is involved. The pseudostratified epithelium is abundant in the lateral wall where the lymphoid-rich fossa of Rosenmüller is located and is a site where NPC tumors most often arises. While the oral epithelium is a site of EBV replication, whether the nasopharyngeal epithelium is a major source of EBV shedding in the nasopharynx is not well defined. Here, we present a 3-dimensional organoid model of the nasopharyngeal pseudostratified epithelium showing that such cells can be infected with EBV in some donor cultures, with examples of both latent and lytic infection. We propose that the cell types of the pseudostratified epithelium should be considered a component of EBV pathogenesis in the nasopharynx and that the difference in donor susceptibility and latent/lytic infection could influence EBV’s fitness in the nasopharynx.

## Introduction

Epstein-Barr virus (EBV) is a human tumor virus from the γ-herpesvirus family [1]. Infection is chronic and mostly asymptomatic but in a subset of individuals, latent infection is associated with different types of B-cell lymphomas and epithelial carcinomas such as nasopharyngeal carcinoma (NPC) [1]. EBV-associated NPC is endemic in Southeast Asia and also occurs with higher incidence in specific populations such as Alaskan Inuits [2]. Diet and host genetics are thought to be risk-factors for NPC but almost all NPC tumors share the characteristic of latent and clonal EBV infection [2]. Thus, it would seem that EBV is not a passenger infection but coincides with the clonal expansion of the neoplastic cell in NPC. EBV immortalizes B-cells; however, there are no reports of immortalization in epithelial cells [3,4]. EBV *in vitro* infection is also inefficient in two-dimensional (2-D) cell culture [5]. Accordingly, many aspects of EBV molecular pathogenesis in epithelial cells is unclear. There is however clear evidence that EBV infection can be detected in preinvasive nasopharyngeal biopsies but in the absence of dysplasia, EBV-infected cells are rarely detected in the normal nasopharyngeal epithelium [5–8]. This infrequency may be due to robust immune surveillance and/or small areas of infection that are difficult to capture by biopsy sampling methods. Thus, studies on EBV molecular pathogenesis in the nasopharynx have relied heavily on cell culture. Conventional 2-D cell culture is used to study EBV latent infection in epithelial cells but it does not reproduce all the cell types of the nasopharyngeal epithelium or capture many aspects of the differentiated biology [3,9]. Furthermore, EBV-infected cell lines in 2-D culture can be refractory to reactivation even when treated with chemical inducers [3,10]. Both latent and lytic infection are thought to encourage the carriage and spread of EBV in the nasopharynx, which presumably would predispose cells to neoplasia by being exposed to EBV infection [2].

Differentiation-induced reactivation in oral stratified keratinocytes cultured in 3-D organotypic rafts explains the lytic pathology of EBV-associated oral hairy leukoplakia [11,12]. The molecular pathogenesis in the nasopharyngeal epithelium is less clear as experimental models of EBV infection in the human nasopharyngeal epithelium have only recently emerged [3,13]. Other than stratified keratinocytes, almost half of the nasopharyngeal epithelium is composed of pseudostratified respiratory epithelium which consists of a variety of cell types (ciliated, mucosecretory, basal and suprabasal) [14]. In this study, we present a *de novo* EBV infection model of the nasopharyngeal pseudostratified epithelium grown in 3-D cell culture from conditionally reprogrammed cells in air-liquid interface (ALI) culture [15–17]. To distinguish this type of pseudostratified ALI culture from other types of ALI culture that model the stratified epithelium (such as organotypic rafts), we herein refer to the pseudostratified ALI model as “pseudo-ALI” culture. Conventionally, pseudo-ALI cultures of airway (bronchial or nasal) epithelial cells are used to study acute virus infections such as influenza virus [18], respiratory syncytial virus [19], rhinovirus [20], and SARS-CoV-2 [21]. Recently, one study has reported that 3-D cultured pseudostratified epithelial cells can indeed be infected by EBV *in vitro* as determined by *in-situ* hybridization of EBV transcripts (EBER1 and BRLF1) using RNAScope [13]. Such sensitive detection methods improve the diagnosis of EBV infection at single cell resolution but the singular detection of EBV transcripts alone cannot distinguish an active EBV (latent/lytic) infection program from an abortive infection in a biologically meaningful manner. It was also not clear whether such EBV infection can be observed in more than one donor or whether the detection of EBV transcripts amounts to virus production. Here, we report that nasopharyngeal pseudo-ALI cultures from different donors can be susceptible to EBV infection. Using primary cells from a collection of 9 donors and EBV molecular diagnostics including immunostaining for EBV latent and lytic proteins, in situ-hybridization for EBERs, EBV genome amplification, and single cell RNA-sequencing (scRNA-seq), examples of both latent and lytic infection are observed. Evidence of donor-specific variation in susceptibility and infection outcome (latent/lytic) is presented. We report that latent infection can occur in such nasopharyngeal pseudo-ALI cultures. These latently-infected cells express cell cycle markers and higher levels of host restriction factors known to limit EBV lytic infection. A third of the transcriptional changes observed in EBV-infected NPC tumors were commonly affected in the latently-infected subcluster of the pseudo-ALI culture, compared to an uninfected pseudo-ALI reference dataset. A third of these commonly perturbed genes (16 genes) were not found in uninfected nasopharyngeal biopsy controls but unique to EBV-infected NPC tumors and the EBV-infected pseudo-ALI culture, most likely attributed to EBV-imposed changes. Given that the pseudo-ALI cultures from some donors were consistently positive for EBERs, but negative for EBV lytic antigens, this is consistent with the hypothesis that while the stratified epithelium produces a lytic infection [12], the pseudostratified epithelium can harbor latently-infected cells.

## Results

### Establishment of a 3-D pseudo-ALI model of *de novo* EBV infection

We have previously demonstrated that conventional ALI culture can reactivate EBV from the NPC cell line, HK1-EBV, producing high infectious titers (>10^6^ infectious green Raji units per cm^2^) [10]. To elucidate EBV pathogenesis in primary cells, a method was developed for *de novo* EBV infection in primary nasopharyngeal cells grown in pseudo-ALI culture. Primary cells from the nasopharynx, at the site of the lymphoid-rich fossa of Rosenmüller, were collected under direct visualization from adult immune-competent donors undergoing endoscopic nasal procedures for reasons other than cancer. Conditionally reprogrammed nasopharyngeal cells were expanded on irradiated mouse 3T3-J2 fibroblasts in the presence of ROCK inhibitor (Y-27632) and lifted to the air-liquid interface on collagen-coated transwell membranes for 4 weeks [15,22]. Once the pseudostratified epithelia have formed in pseudo-ALI culture, EBV inoculum was applied to the apical surface by co-culture with the EBV-positive Akata cell line that have been reactivated with anti-human IgG. The producer Akata cell line is recombinantly-infected with EBV expressing neomycin resistance and the EGFP marker gene inserted into the non-essential *BXLF1*, herein referred to as rAkata [23]. As mock control, target cells were co-cultured with EBV-negative Akata cells similarly treated with anti-human IgG antibody.

**Fig 1:**
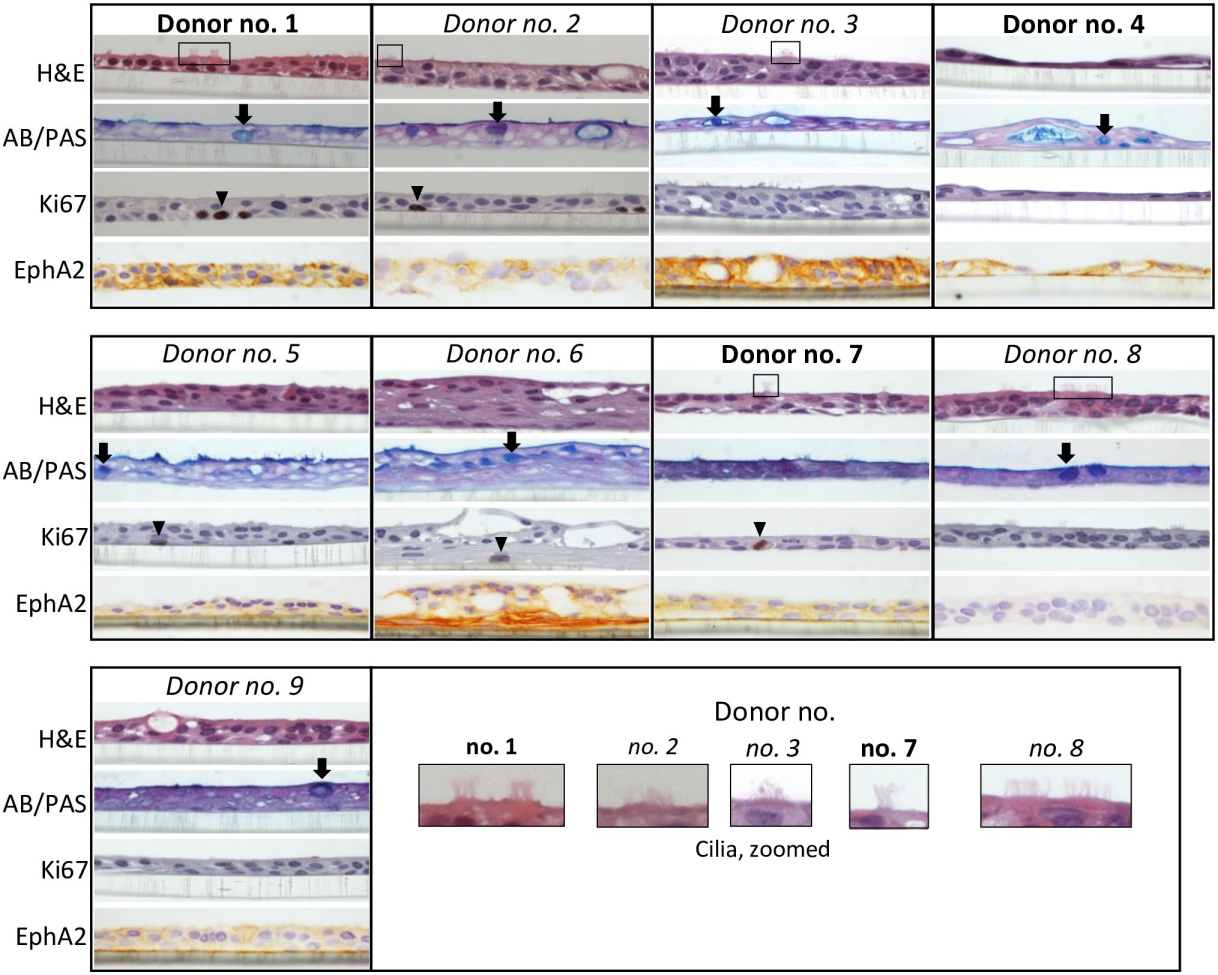
Histological analysis of pseudo-ALI cultures derived from the nasopharynx for markers of cellular differentiation and the EBV epithelial cell receptor. Shown are pseudo-ALI cultures susceptible (bold) and non-susceptible (italics) to *de novo* EBV infection. Examples of ciliated cells are marked by a black box in the Hematoxylin & Eosin (H&E) stain. Mucin-producing cells are marked by a black arrow in the Alcian blue/periodic acid Schiff (AB/PAS) stain. Basal cells that are proliferating are marked by a black arrowhead in the Ki67 stain. Expression of the EBV epithelial cell receptor, Ephrin type-A receptor 2 (EphA2), is indicated by brown 3, 3’–diaminobenzidine (DAB) staining counterstained with nuclear blue.

**Fig 2:**
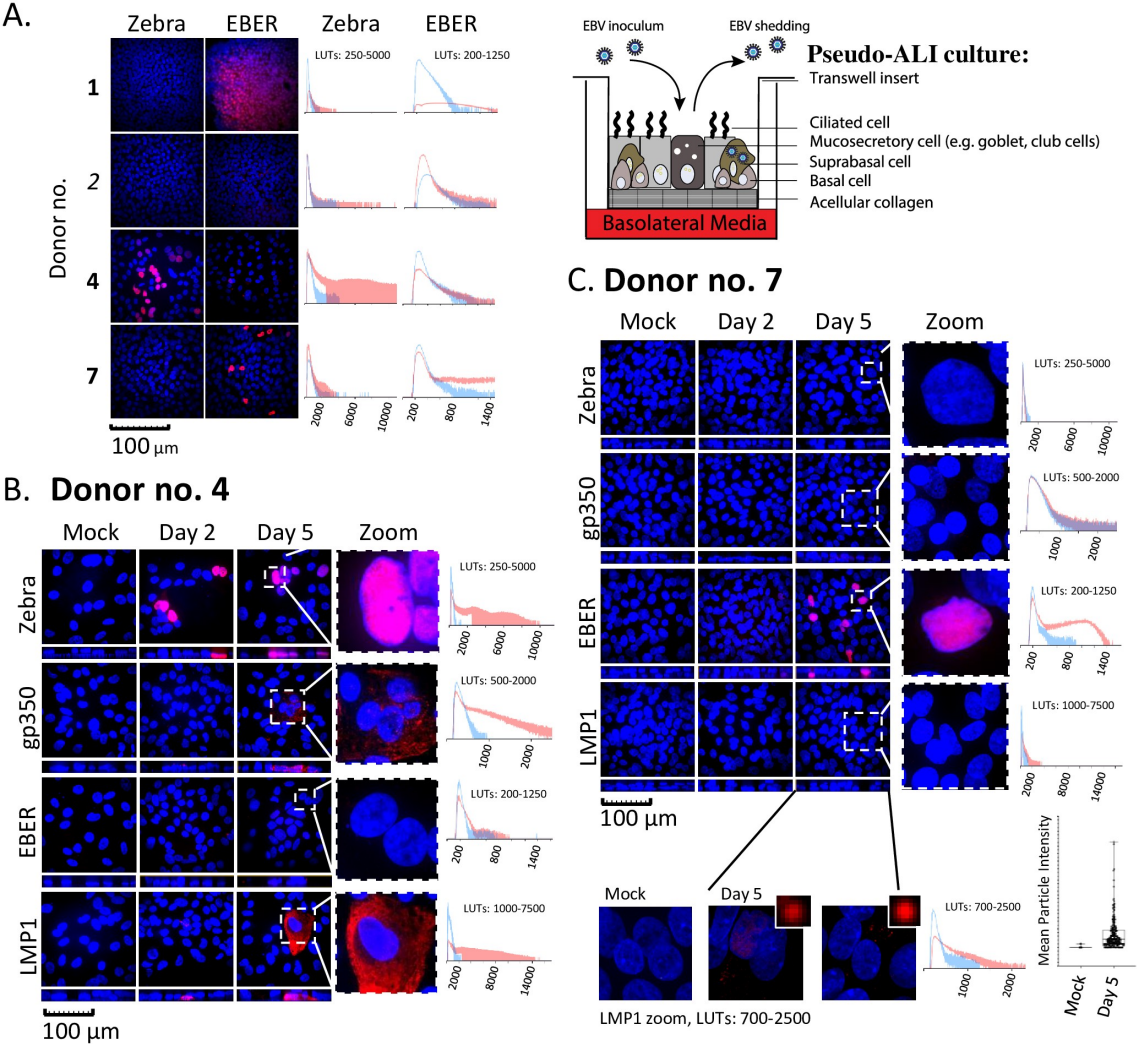
EBV *de novo* infection of primary nasopharynx-derived epithelial cells in pseudo-ALI culture. A-C, Immunofluorescence staining and EBER-ISH images (red) for EBV molecular diagnostics in pseudo-ALI cultures infected with EBV-positive rAkata B-cells or EBV-negative Akata B-cells (mock), counterstained with DAPI (blue). Shown are maximum intensity projections of confocal images on the *xy* (square) and *xz* (rectangle) planes. The pixel signal intensity of the EBV markers in the unzoomed image, in mock (blue line) and EBV-infected (red line) samples, are compared in the histograms. (A) Shown are the results for four donors. Cells were harvested at days 4–5 p.i. for Zebra and days 5–7 p.i. for EBER-ISH. More extensive analysis at days 2 and 5 p.i. was performed for donors (B) no. 4 and (C) no. 7. LMP1 foci in the weakly stained sample (donor no. 7) was also scored as mean particle intensity, from the unzoomed image. Bold, susceptible donor sample; italics, non-susceptible donor sample. LUTs, look-up-tables.

Cells differentiated in pseudo-ALI culture were analyzed by histopathology to control for differentiation into pseudostratified epithelium (Fig 1). Hematoxylin and eosin stain demonstrated the presence of pseudostratified epithelium and ciliated cells. There were cultures from some donors with thinner (donor no. 4) or thicker (donor no. 6) epithelium but overall the histology is consistent with a pseudostratified, but not a stratified epithelium. Alcian blue and periodic acid Schiff stain stained positively for mucin-secreting cells. Furthermore, immunohistochemistry staining for the proliferation marker Ki67, showed the infrequent presence of cycling cells demarcating the basal layer. To identify susceptible samples, EBV molecular diagnostics for latent and lytic markers of infection were developed for whole-mount staining of pseudo-ALI culture. These molecular diagnostics were first validated in the HK1-EBV cell line, in which 2-D culture is latent but 3-D ALI culture triggers lytic reactivation [10]. EBERs are abundantly expressed during latent infection and is diagnostic of EBV infection in NPC tumors, but strongly downregulated during lytic infection [24–27]. Based on this latent/lytic cycle-dependent expression, the strong presence of EBERs (detected in the nucleus) and absent detection of EBV lytic antigens is diagnostic of latent infection. As exemplified in HK1-EBV cells maintained in monolayer culture, such latently-infected cells stained positively for EBERs by *in situ* hybridization (EBER-ISH) in the nucleus, but negative for Zebra (immediate-early protein) in the nucleus and negative for gp350 (late glycoprotein) in the cytoplasm (S1 Fig). In contrast, HK1-EBV cells reactivated by ALI culture stained negatively for EBERs but positively for Zebra and gp350 (S1 Fig). Staining for the EBV oncoprotein, LMP1, identifies both latent and lytic infection [10,28]. Stained images are scored by pixel intensity represented as a histogram compared to the mock (Fig 2). Punctate LMP1 foci can also be discriminated as particles and scored by particle intensity, represented as a box and whisker plot (Fig 2C).

### EBV infection in pseudo-ALI culture show variation in donor susceptibility

Both susceptible and non-susceptible cultures were identified by EBV molecular diagnostics (Table 1). A total of 3 pseudo-ALI cultures (donor no. 1, 4, 7) were susceptible to EBV infection, while cultures from the other 6 donors were negative for the tested EBV molecular markers (Fig 2A and S2 Fig). Pseudo-ALI cultures from 2 donors (nos. 1 and 7) were positive for markers of latent infection, while cultures from donor no. 4 were positive for markers of lytic infection (Fig 2A–2C and Table 1). Stitched images showed no evidence of residual B-cell contamination from the inoculum after extensive washing before fixing and processing for stains (S3 Fig). In some cases, susceptible and non-susceptible cultures could be identified in the same experiment using the same stock of inoculum (Table 1). Thus, a failure to infect was due to donor-specific variation not due to experimental variation. Infections were repeated on low-passaged cells thawed from banked nasopharyngeal conditionally reprogrammed cells. In almost all cases of biological repeats (53 out of 54), either from susceptible (donor no. 4 and 7) or non-susceptible (donor no. 3, 5 and 8) donors, the same result in donor-specific susceptibility and latent/lytic profiles were observed (Table 1, parentheses). Susceptibility to EBV did not appear to correlate with the presence or absence of comorbidity, although the number of samples collected is too small for statistical analysis. Despite the fact that donors were consented at the time of surgery for rhinosinusitis or other sinus conditions, all donors had seemingly normal (or sub-clinical) presentation of the nasopharynx. Furthermore, since the pseudo-ALI cultures are grown in the presence of antibiotics and antimycotics, any difference in EBV infection is likely attributed to differences in host genetics, epigenetics or possibly the virome, rather than the bacterial or fungal microbiome. The EBV entry receptor for epithelial cells, Ephrin receptor A2 (EphA2) [29,30], was detected on the plasma membrane in all susceptible pseudo-ALI cultures but some of the cultures from non-susceptible samples were also strongly positive for EphA2, exemplified by donors no. 3 and 6 (Fig 1 and Table 1). This indicates that while expression of EphA2 is consistent with EBV infection, other donor-dependent restriction factors are likely involved.

**Table 1:** Summary of molecular diagnostic results from EBV infection of pseudo-ALIs.

Donor No.* (reference code)	Experiment No.	Susceptibility to EBV (-, +)	EphA2**	Latent	Lytic (immediate-early, late)		Latent-lytic	Reason for surgery/comorbidity [other]
				EBER (-/+)	Zebra (-/+)	gp350 (-/+)	LMP1 (-/+)	
1 (H04)	1	+	++	+	-	n/a	n/a	CRSsNP***/none
2 (H05)		-	+	-	-	n/a	n/a	Fungus ball, septal deviation, allergic rhinitis/inflammatory polyarthropathy
3 (H08)	2	-	+++	- (6/6)	- (6/6)	n/a	n/a	CRSsNP/inflammatory polyarthropathy, Yellow Nail Syndrome
4 (H10)	3	+	++	-	+ (5/6)	+	+	Left turbinate hypertrophy/none
5 (H12)	4	-	+	- (6/6)	- (6/6)	-	-	CRSsNP/inflammatory polyarthropathy
6 (H13)		-	+++	-	-	-	-	Silent sinus syndrome/thyroid disorder-unspecified, [Crohn’s disease]
7 (H15)	5	+	++	+ (6/6)	- (6/6)	-	+ weak	Septal perforation-unknown etiology/[rheumatoid arthritis]
8 (H17)	6	-	-	- (6/6)	- (6/6)	-	-	Septal deviation/none
9 (H18)		-	+	-	-	-	-	Odontogenic sinusitis and septal deviation/none

*Donor cells were obtained from the nasopharynx. The reference code denotes a unique donor identifier;

**Scoring criteria: -,+,++,+++;

***CRSsNP, Chronic rhinosinusitis without nasal polyps; n/a, not available; biological repeats are indicated in parentheses.

**Fig 3:**
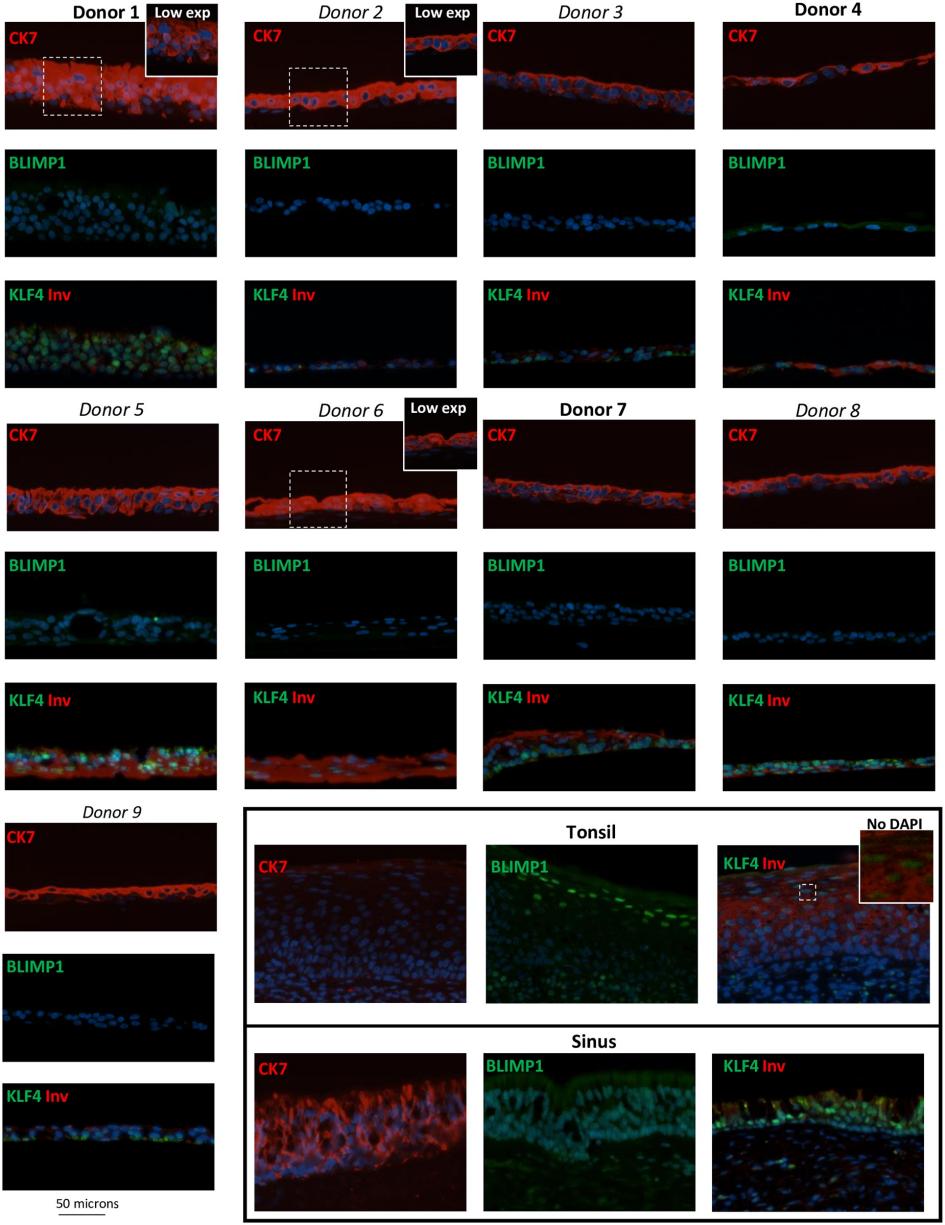
Immunostaining for markers of the pseudostratified epithelium and cellular differentiation factors. Immunofluorescence images of FFPE sections from pseudo-ALI cultures stained for the pseudostratified epithelial marker (CK7) and markers of cellular differentiation (BLIMP1, KLF4 and Inv). Sinus and tonsil sections served as control tissue for the pseudostratified and squamous epithelium, respectively. CK7, cytokeratin 7; Inv, involucrin; Low exp, low exposure.

**Fig 4:**
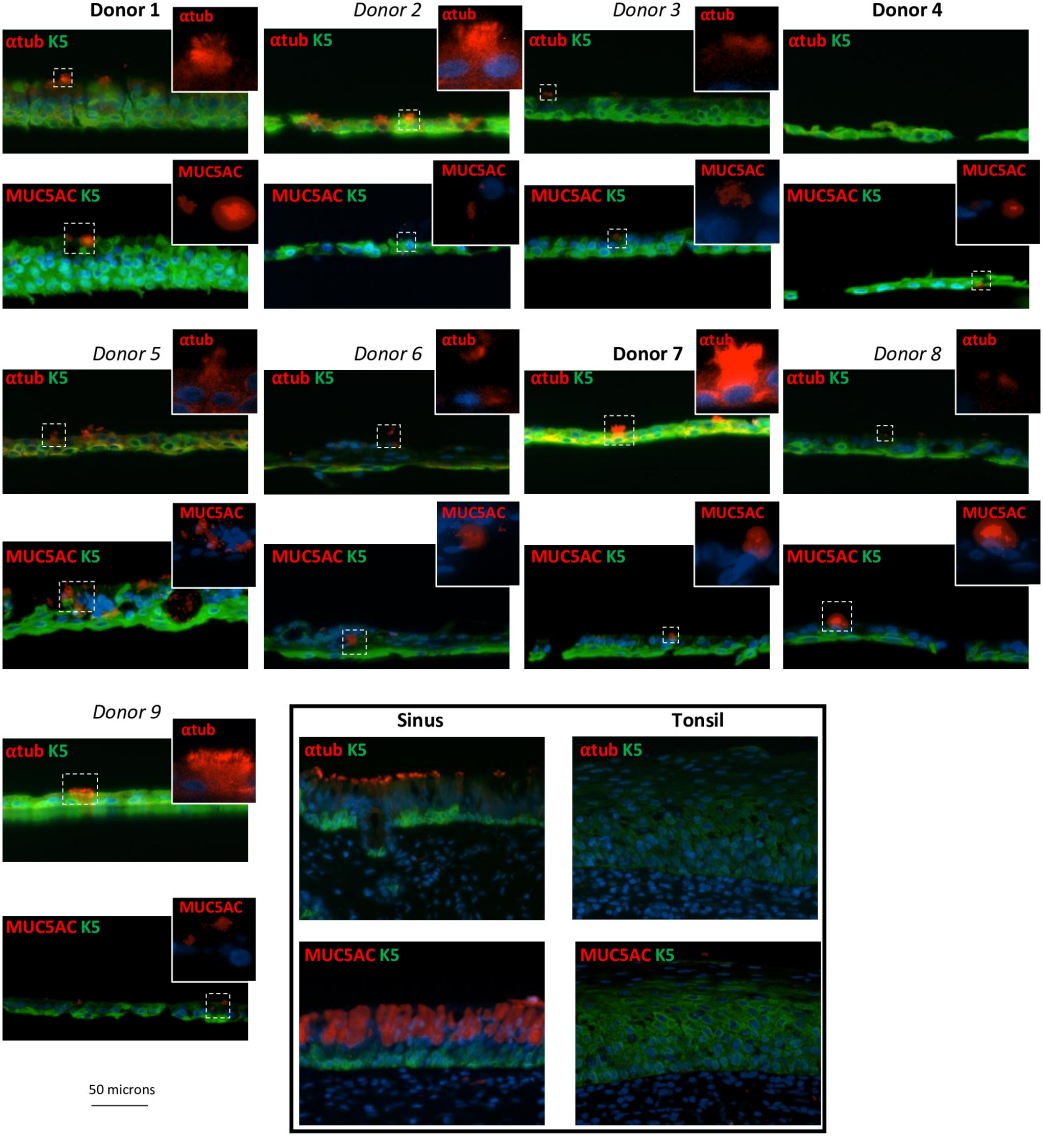
Immunostaining for cell-type specific markers unique associated with the pseudostratified epithelium. Immunofluorescence images of FFPE sections from pseudo-ALI cultures stained for cilia (α-tubulin) and for a mucosecretory Goblet cell marker (MUC5AC). Sinus and tonsil sections served as positive and negative tissue controls, respectively. K5, keratin 5.

**Fig 5:**
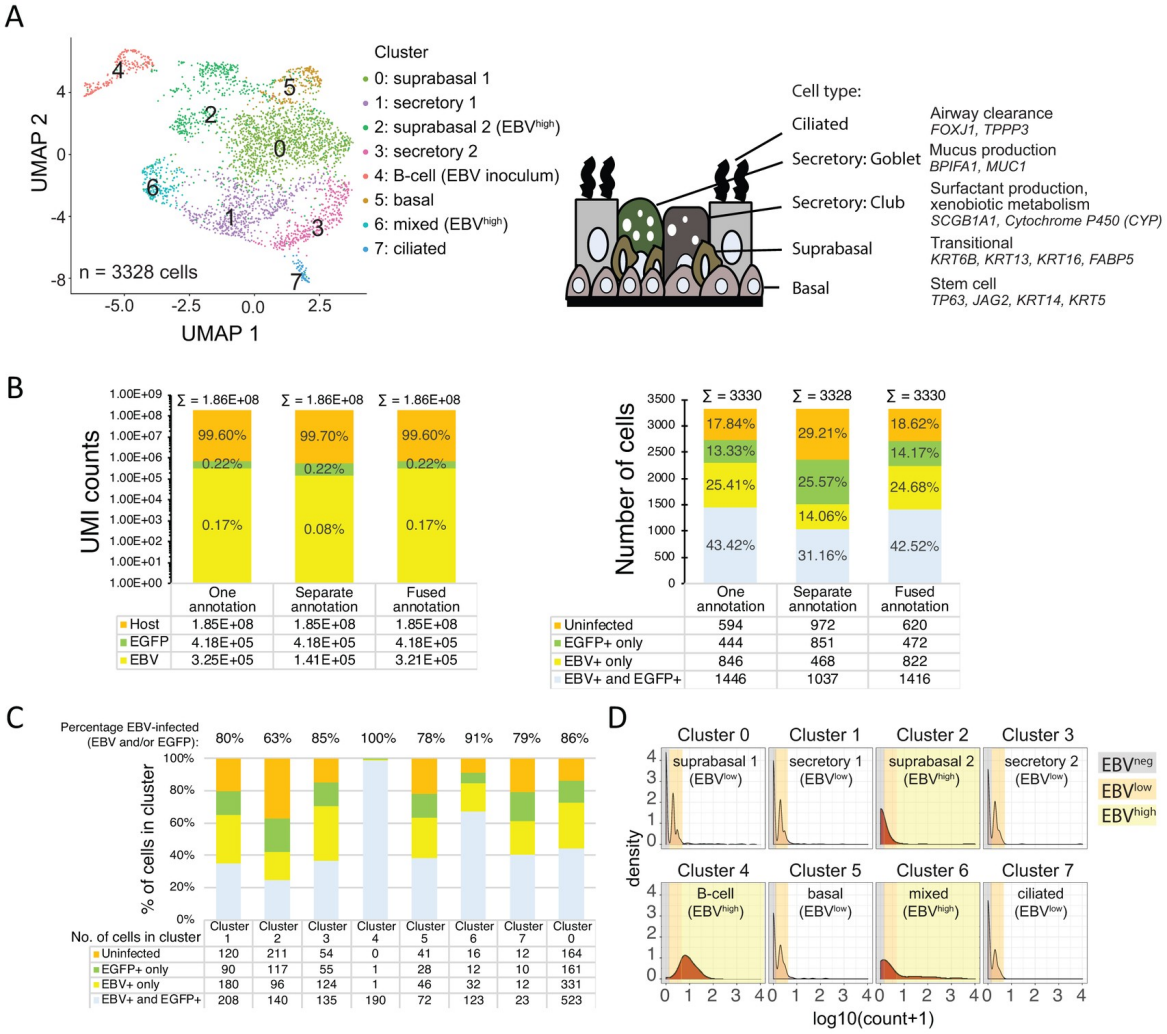
Identification of EBV-infected cell types from scRNA-seq performed on a pseudo-ALI culture from donor no. 4. (A) Uniform Manifold Approximation and Projection (UMAP) plots displaying the major cell clusters and assigned identity from host marker genes. Schematic displays the cell types in the pseudostratified nasal epithelium that are represented in the cell clusters. (B) Comparison of alignment methods against different EBV genome annotations by UMI counts and numbers of cells. (C) The percentage of EBV-infected cells is displayed for each cluster. Shown are the results from alignment to the fused annotation EBV genome, (D) Density plots of log10 transformed pseudocount (UMI count+1) for EBV reads against normalized cell numbers displayed for each cell cluster. Cell numbers are normalized for the total number of cells in each cluster. Cells are grouped by EBV^negative^, EBV^low^ and EBV^high^ populations designated by reads aligning to the EBV genome. Density plots with a similar profile are similarly colored. Where applicable (B-C) cells with reads aligning to the EGFP gene is also shown.

A thorough histological examination of cellular markers that define the pseudostratified epithelium did not differ between the donor pseudo-ALI cultures. All pseudo-ALI cultures stained strongly for cytokeratin 7 (CK7) which marked the columnar cells in pseudostratified sinus tissue but not the tonsillar squamous epithelium (Fig 3) [31]. Furthermore, cilia marked by α-tubulin and the mucosecretory Goblet cells marked by MUC5AC staining, were identified in the pseudo-ALI cultures and sinus tissue but not the tonsillar squamous tissue control (Fig 4). These results are consistent with the pseudo-ALI cultures being histologically similar to the pseudostratified epithelium *in vivo*. The exception was donor no. 4 in which ciliated cells was not observed by staining formalin-fixed paraffin-embedded (FFPE) sections, which sometimes can be afflicted by tangential cuts, but could be identified as a distinct cell cluster in subsequent scRNA-seq analysis (Fig 5). The cellular differentiation-dependent transcription factors BLIMP1 and KLF4 previously associated with EBV lytic reactivation also did not distinguish the donor pseudo-ALI cultures [32]. The differentiated cells of the tonsillar stratified epithelium, but not the pseudostratified epithelium from sinus tissue or pseudo-ALI culture, stained positively for nuclear BLIMP1 (Fig 3). Nuclear staining of KLF4 was more strongly detected in the pseudostratified epithelium of sinus tissue than the stratified tonsillar epithelium, but its detection in pseudo-ALI cultures was irrespective of EBV susceptibility and not unique to the donor pseudo-ALI culture (donor 4) that yielded lytic-infected cells (Fig 3). Additionally, involucrin which strongly stained multiple apical layers of the tonsillar squamous epithelium but weakly stained the pseudostratified sinus, appeared in the pseudo-ALI culture of donor no. 4 (Fig 3). However, involucrin also appeared in the pseudo-ALI culture of donor no. 7 (Fig 3) which lacks any sign of lytic infection (Fig 2A and 2C). These results indicate that the known differentiation markers associated with lytic infection do not explicitly explain the type of EBV infection (latent or lytic) observed in pseudo-ALI cultures, which may be affected by the expression of other lytic restriction factors such as type I interferon genes [33]. Unfortunately, it was not possible to stain FFPE sections of EBV-infected cells for cellular and EBV markers because the cultures were too disrupted for intact FFPE sectioning and staining. There was also not sufficient primary cell material to generate enough pseudo-ALI cultures for whole-mount staining of all these cellular and EBV markers. However, these results of mock-infected controls do indicate that the pseudo-ALI cultures represent the cell types of the pseudostratified epithelium observed *in vivo*. Complementary approaches such as scRNA-seq could help to distinguish EBV infection by cell type and to elucidate host determinants of EBV infection.

### Molecular diagnosis of EBV infection reveals donor-specific differences in molecular pathogenesis—Latent versus lytic infection

Samples from donors no. 4 and 7 were subjected to more extensive analyses at days 2 and 5 post-infection (p.i.). Donor sample no. 4 stained positive for Zebra and LMP1 beginning at day 2 p.i., followed by gp350 at day 5 p.i., denoting a lytic infection (Fig 2B). Donor sample no. 7 showed positivity for EBERs at day 5 p.i., denoting a latent infection (Fig 2C). For donor sample no. 4, EBV replication was measured by quantitative PCR of DNA harvested from extracellular or cell-associated DNase-resistant encapsidated virus (Table 2). As input control, pseudo-ALI cultures were fixed before co-culture with the inoculum. While the EBV genome copy number in the input control did not increase from day 2 to 5 p.i., extracellular EBV increased 37-fold (3.13 x 10^4^ copies at day 2 p.i. to 1.16 x 10^6^ copies at day 5 p.i.). EBV copy numbers did not increase in the cell-associated virus which measured between 1.55–4.06 x 10^4^ copies. This indicates that the majority of encapsidated EBV are packaged for secretion. Using virus collected from the extracellular source, infectious units were scored by the Green Raji Unit (GRU) assay in the non-producer Raji cell line. The secreted virus is indeed infectious, reaching 1.07 x 10^5^ GRUs by day 5 p.i. (Table 2).

**Table 2:** Summary of EBV quantitative PCR and infectious titer results for pseudo-ALI cultures from donor no.4.

Condition (days p.i.)	EBV DNA quantitative PCR (n = 3)						Green Raji Unit [GRU] titer (n = 2)	
	Extracellular EBV genomes per ALI			Cell-associated EBV genomes per ALI			GRU per ALI	S.D.
	CT	Copy Number	S.D.	CT	Copy Number	S.D.		
Day 2, rAkata-infected (n = 2)	28.84±0.08	3.13E+04	1.87E+03	28.56±0.12	4.06E+04	3.25E+03	624	238
Day 5, rAkata-infected (n = 6)	23.90±0.07	1.16E+06	4.80E+04	30.23±0.31	1.55E+04	2.62E+03	1.07E+05	6.29E+04
Day 2, input control (n = 2)	29.38±0.07	2.16E+04	1.27E+03	30.08±0.12	1.21E+04	1.28E+03	0	0
Day 5, input control (n = 3)	29.46±0.15	2.00E+04	2.44E+03	31.40±0.34	4.67E+03	1.58E+03	8	8
No template or media control	33.58±0.30	0	0	32.06±0.13	0	0	0	0

### Single cell RNA-sequencing reveals cell type-specific EBV transcriptional profiles

**Fig 6:**
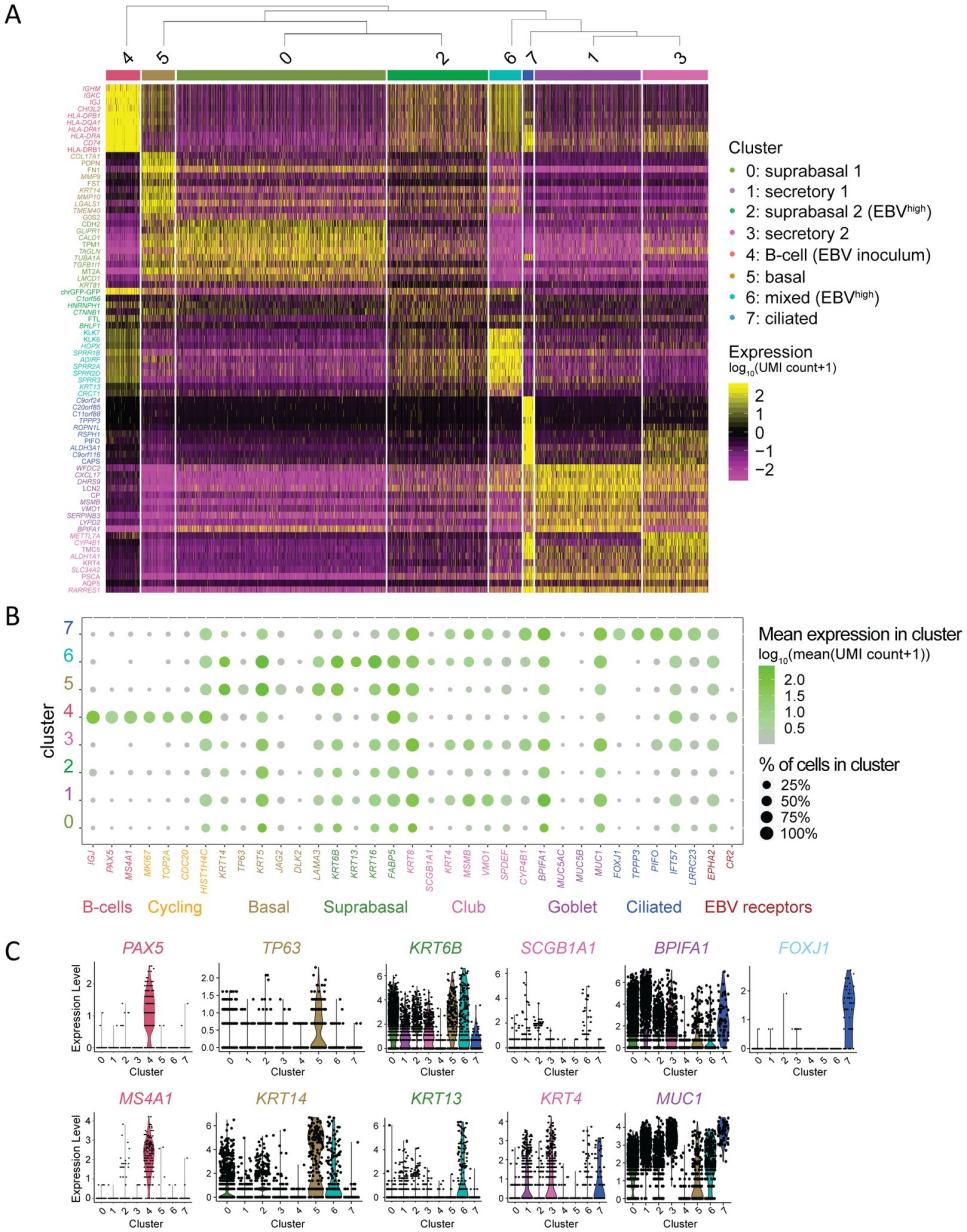
Assignment of cluster identity by cell type-specific marker genes for donor no. 4. (A) Hierarchical clustering of heatmap representing cluster-specific top marker genes. Expression shows log10 transformed pseudocount (UMI count+1). (B) Dot plot representing marker gene expression by cluster. Dot size indicates the percentage of cells in each cluster expressing the marker gene. Color gradient indicates the mean expression of each marker gene, averaged from positively scored cells, for each cluster. Cluster identity enriched for cell-type specific marker genes are color-coded. Cluster 6 expresses a mixture of marker genes indicative of multiple cell types (basal, suprabasal, goblet/mucosecretory). The EBV receptor gene, *CR1*, is not shown as it was not expressed according to the count matrix. (C) A few of the highly cell type-specific marker genes were chosen for display by violin plots.

scRNA-seq analysis poses a challenge for all herpesvirus genomes because of overlapping 3’ co-terminal herpesvirus transcripts, whose non-uniquely mapped reads are discarded in the 10X Genomics single cell analysis pipeline [34]. We reasoned that this bioinformatics challenge is theoretically possible with the EBV γ-herpesvirus genome given that it has been demonstrated for α- and β-herpesviruses [35–37]. Recent reports have demonstrated that scRNA-seq alignment to EBV is possible in EBV-infected lymphoblastoid cell lines and NPC tumors [38–40], although alignment spanning the EBV genome has yet to be demonstrated. This is more likely to be observed in lytic-infected cells such as those in pseudo-ALI culture. To identify EBV-infected cell types, the pseudo-ALI culture at day 4 p.i. from donor sample no. 4 was subjected to scRNA-seq. Cell clusters (Fig 5A) were assigned cell identities using *a priori-*defined marker genes (Fig 6) established from primary human nasal epithelial cells grown in pseudo-ALI culture [41] as well as from primary nasal tissue (The Human Cell Atlas Lung Consortium) [42]. All major airway epithelial cell types (basal, mucosecretory, suprabasal and ciliated) could be identified (Fig 5A). In order to improve alignment to the partially annotated EBV genome (NCBI KC207813.1), the Akata strain reference genome was updated with additional exon annotation totaling 87 genes. In order to identify the optimal alignment, we tested several algorithms using the 10X Genomics Cell Ranger pipeline. The reads were either aligned to the whole EBV genome as one annotation, as separately annotated genes, or as annotated genes but with genes that have regions of overlap in the same direction represented as fusion genes. Alignment to the separate annotation assigns the identity of EBV transcripts according to the reference annotations, but alignment to the other two annotations counts more EBV reads. Overall, the EBV transcriptome represents 0.08% (separate annotation) to 0.17% (one annotation and fused annotation) of the total transcriptome (Fig 5B). This is similar to estimates from bulk RNA-seq of lymphoblastoid cell lines carrying latent EBV, where the majority of samples had EBV reads measuring 0.1–0.5% of the total transcriptome [43]. A large majority of the cells (71%-82%) expressed EBV and/or EGFP transcripts (Fig 5B). It is noteworthy that an inherent methodological limitation of the single-cell sequencing technology is its limited capture rate, in our case approximately 30–32% of total mRNA. Thus, it is to be expected that a significant proportion of the infected cells are false negative for EBV or EGFP transcripts. Also, the captured EGFP or EBV reads may only reflect a small portion of the expressed reads from the virus. Thus, although in theory infected cells would express both EGFP and EBV transcripts, in practice there was a small number of cells with low numbers of EBV reads that had no EGFP reads, and vice versa (S4 Fig). There were also cells that had mid-level EGFP reads but no EBV reads that are likely to have resulted from abortive infection (S4 Fig). Nonetheless, the overall number of EBV reads was positively correlated with the number of EGFP reads (S4 Fig).

**Fig 7:**
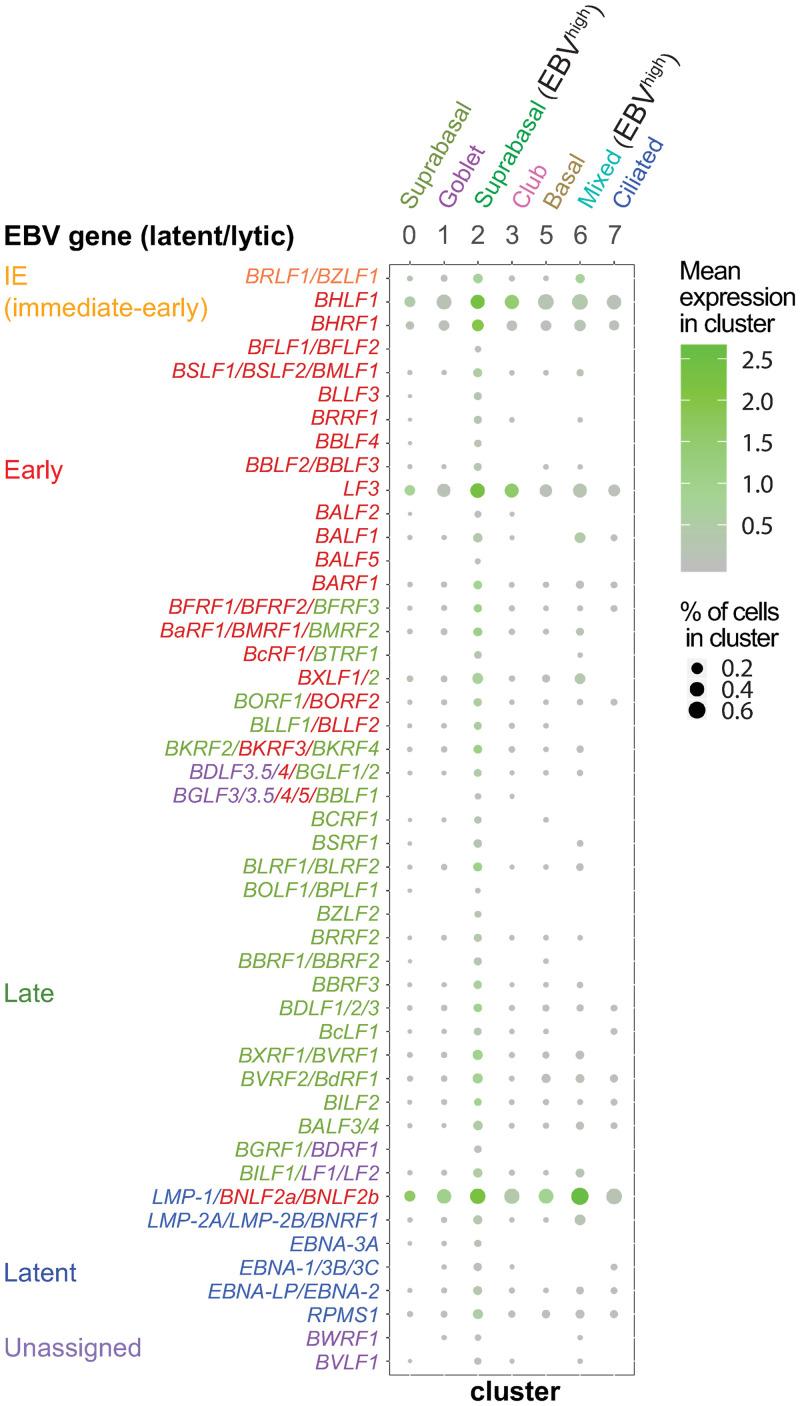
Averaged expression of EBV genes by cluster. Dot plot shows expression of EBV genes for epithelial cell clusters in the EBV-infected pseudo-ALI culture of donor no. 4. Genes are grouped in latent or lytic (immediate-early/early/late). Unassigned genes are in purple. Mean expression by cluster shows log10 transformed pseudocount (UMI count+1) for EBV gene averaged by cluster. Dot size indicates the percentage of cells in each cluster expressing the EBV gene. The scRNA-seq reads were aligned to the EBV genome fused annotation.

Every cluster scored positive for EBV and/or EGFP reads (Fig 5C). *BHLF1*, *BHRF1*, *LF3 and LMP1/BNLF2a/BNLF2b* were the most frequently detected genes in the highest proportion of epithelial cells across clusters (Fig 7). While the B-cell inoculum was not detectable by immunofluorescence staining because it was subjected to extensive washing before fixation to remove serum contaminants, it was however detected as a distinct cluster by scRNA-seq where the cells were subjected to a gentler wash in serum-containing buffer to preserve cell viability for optimum scRNA-seq processing. All the cells in cluster 4 defined as the B-cell inoculum by B-cell markers (*PAX5*, *MS4A1*), expressed EBV and/or EGFP transcripts, with >97% of cells showing both EBV and EGFP (Fig 5C). Across the epithelial cell clusters (clusters 1, 2, 3, 5, 6, 7, 0) the percent of cells with EBV reads ranged between 63%-91%, with no clear difference in susceptibility between clusters (Fig 5C). However, density plots revealed two distinct EBV expression profiles, clusters with a peak at low UMI count (log_10_(count+1) < 0.3, clusters 0, 1, 3, 5, 7) denoted as EBV^low^, and clusters with 1–3 log_10_ higher UMI counts (clusters 2, 4, 6) denoted as EBV^high^ (Fig 5D).

### Lytic infection is confined to suprabasal cells while latent infection appears in basal/mucosecretory and ciliated cell types

**Fig 8:**
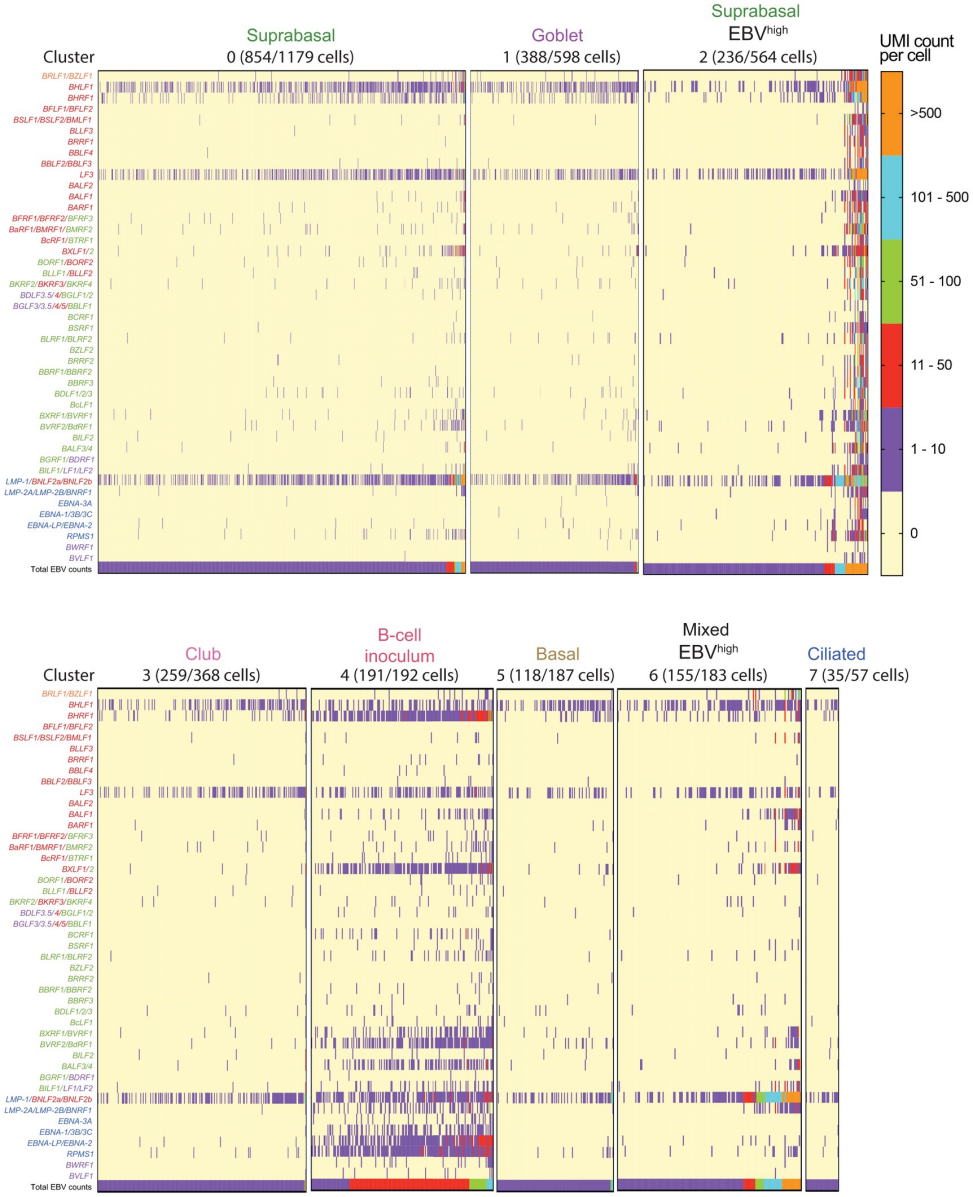
Distinguishing EBV gene expression profiles by cell type. Heatmaps show EBV gene expression by UMI count per cell, displayed for each cluster, for the EBV-infected pseudo-ALI culture of donor no. 4. To distinguish EBV^high^ from EBV^low^ cells, total EBV counts (total EBV UMI counts per cell) are shown at the bottom of each heatmap. Only the EBV-infected cells (with reads aligning to the EBV genome) are shown with the number of EBV-infected cells displayed for each cluster indicated in parenthesis. EBV genes are color-coded into lytic: immediate-early (orange)/early (red)/late (green); latent (blue); and unassigned (purple). The scRNA-seq reads were aligned to the EBV genome fused annotation.

**Fig 9:**
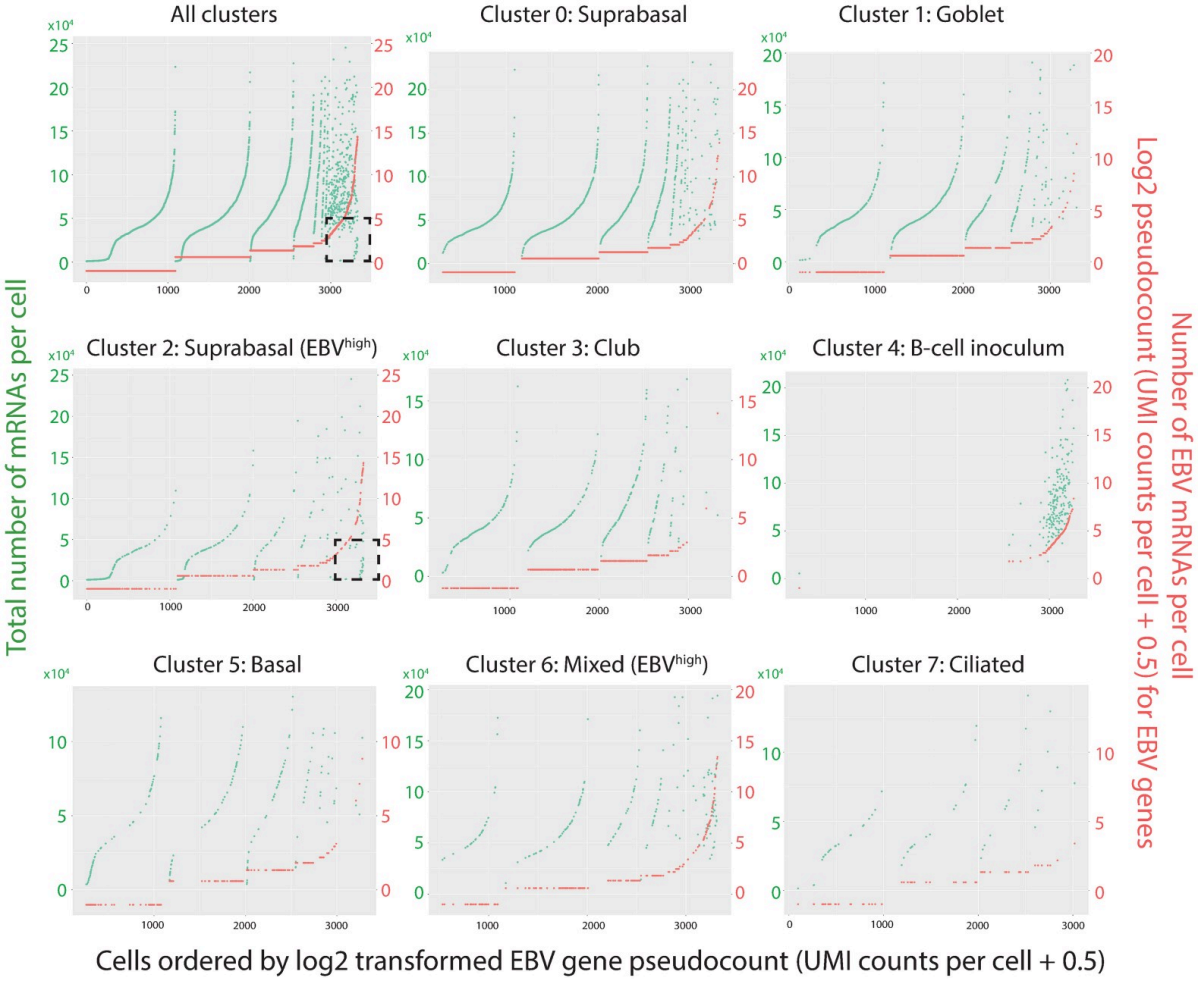
Comparison of EBV gene count to total number of mRNAs detected per cell. Shown are the results for the EBV-infected pseudo-ALI culture from donor no. 4. Dot plots show EBV gene pseudocount (red) and the total number of mRNAs (green) per cell. Each dot represents one cell. Cells are ordered by the EBV gene pseudocount on the x-axes. Data are organized by cluster. Dashed black box, denotes cells with high numbers of EBV transcripts (or mRNA molecules) but low total (host and viral) transcript counts representative of host shut-off. Only cells with >1000 mRNAs per cell are shown as defined by the filtering criteria (see S1 Supplementary Methods).

EBV^low^ cells found in all clusters displayed a distinct expression pattern (*BHLF1*, *BHRF1*, *LF3*, and the fused annotation *LMP-1/BNLF2a/BNLF2b*) which did not resemble a canonical type I/II/III latency profile (Fig 8). These cells are likely to be latent, refractory or in the early stage of the lytic cascade. These EBV^low^ cells are predominantly found in basal, mucosecretory and ciliated cell types but also in a group of suprabasal cells defined by cluster 0 (Fig 8). The mixed cell types in cluster 6 could be further divided into 4 sub-populations with distinct marker gene expression (S5A Fig). EBV^high^ cells within subcluster 6–4 with basal cell features (S5 Fig) and the EBV^high^ suprabasal cells in cluster 0 (Fig 8) display high levels of *BZLF1/BRLF1* indicative of reactivation. Lytic infection was mainly observed in EBV^high^ suprabasal cells (cluster 2) where there is global induction of EBV genes (Fig 8) but shut-off of host mRNA (Fig 9). Conservative thresholds (as defined by the number of mapped EBV genes per cell and the percentage of transcripts mapped to EBV) were introduced in order to define cells by lytic or latent infection in cluster 2 and thereby remove cells with intermediate features which may be in a transitional stage (S6A Fig). Such criteria agreed with the expected expression of *BRLF1/BZLF1* in lytic cells (S6B Fig). In total 22 of 236 EBV-infected cells in cluster 2 were designated lytic and 171 of 236 EBV-infected cells in cluster 2 were considered to be strictly latent (denoted EBV^latentlow^). Overall, all cell types in the pseudo-ALI were susceptible to EBV-infection; however productive virus infection as defined by global induction of EBV genes was mainly confined to suprabasal cells. Despite the concerns of overlapping and low abundance viral transcripts evading capture by scRNA-seq [34], we conclude that with the appropriate reference annotation and careful assignment of threshold criteria, EBV latent and lytic infection can be denoted by scRNA-seq.

### EBV expression in pseudo-ALI display similarities to primary NPC

We further investigated if the EBV gene expression in the infected pseudo-ALI were comparable with primary NPC tissue [39]. The publicly available scRNA-seq data from three NPC tumors (NPC36, 46, 50) from the study by Jin S. *et al*. 2020 [39] had the highest fraction of reads in epithelial cells aligning with EBV (S1 Table) and were selected for further analysis. Two of the three tumors, NPC36 and NPC50 had equivalent EBV normalized reads (counts per million, cpm) as the EBV^latentlow^ cells in cluster 2 (S1 Table). Similar to the pseudo-ALI, expression of the EBV genes were primarily from the merged *LMP1/BNLF2a/BNLF2b* gene annotation in these three NPC tumors (S7 Fig). However, *BHRF1*, *BHLF1* and *LF3* were absent in all NPC cells. It is possible that expression of protein encoding genes may be better tolerated in the pseudo-ALI culture compared with a tumor microenvironment subjected to immunological pressure. No lytic cells were detected in the NPC cells, although the total number of EBV-positive epithelial cells were low (n = 589) in these datasets.

Host gene perturbation in the pseudo-ALI cells were compared to identify markers that would distinguish the different EBV^latentlow^/EBV^latenthigh^/EBV^lytic^ infection states. The EBV- and EGFP-negative pseudo-ALI cells were expected to have false negative/EBV-positive cells due to the low capture rate of single-cell sequencing. A comparison was made between EBV^latenthigh^ and EBV^latentlow^ cells from cluster 2 which revealed that five genes were significantly upregulated in the EBV^latenthigh^ cells compared with EBV^latentlow^ cells (S2 Table). Three (*APOBEC3A*, *IL36G* and *S100A7*) of the five genes are associated with immune response to microbes, likely induced by the higher levels of EBV gene expression. These three genes and *KLK5* were also significantly upregulated in EBV^latenthigh^ cells in all epithelial clusters (S2 Table). In stark contrast, comparison of EBV^latentlow^ and EBV^lytic^ cells generated a list of 4130 perturbed genes. This large number of perturbed genes is consistent with the global effect that EBV reactivation has on the host transcriptome in lytic cells.

**Fig 10:**
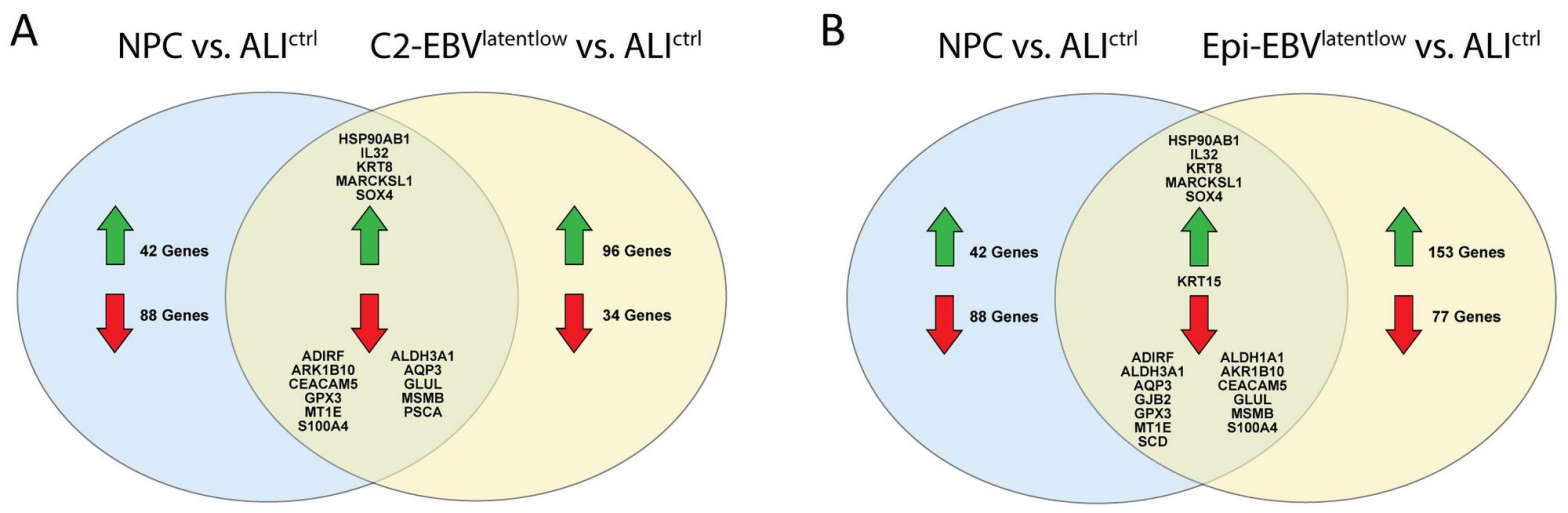
Venn diagram of differentially expressed genes. (A, B) NPC tumors (NPC36, NPC46 and NPC50, [39]), (A) EBV^latentlow^ cells from cluster 2 (C2-EBV^latentlow^), and (B) EBV^latentlow^ cells from all epithelial clusters (Epi-EBV^latentlow^) in the EBV-infected pseudo-ALI culture from donor no. 4 were compared against an uninfected pseudo-ALI culture from [41] as reference control (ALI^ctrl^).

In order to examine whether the changes in the transcriptome caused by EBV-infection in our nasopharyngeal pseudo-ALI show similarities with NPC tumors [39], we compared both datasets with scRNA-seq data from an uninfected pseudo-ALI culture [41]. The EBV^latentlow^ cells from cluster 2 was used to represent latently-infected cells in the pseudo-ALI culture. Highly similar results were obtained when using the EBV^latentlow^ cells from all epithelial clusters (S2 Table). Forty-eight genes (approximately a third of all differentially-expressed genes) were perturbed in both the NPC and cluster 2 EBV^latentlow^ datasets compared with the uninfected pseudo-ALI (S2 Table). An additional uninfected dataset from non-tumor nasopharyngeal biopsies [39] was used to filter out genes which were differentially expressed irrespective of EBV infection. Of the 48 perturbed genes, 32 were found in this control analysis. The remaining 16 genes were commonly perturbed in NPC and cluster 2 EBV^latentlow^ pseudo-ALI cells compared with uninfected pseudo-ALI and therefore likely attributed to EBV-infection (Fig 10A). Additional three genes were commonly downregulated with NPC when using EBV^latentlow^ pseudo-ALI cells from all epithelial cell clusters compared with the uninfected pseudo-ALI (Fig 10B and S2 Table).

### Gene expression profiles of cellular differentiation, EBV attachment, restriction factors and cell cycle

The expression levels of the epithelial differentiation markers *IVL*, *PRDM1* (*BLIMP1*), *KLF4*, *SCEL*, *SPRR1* and *SPRR1B* were analyzed between the pseudo-ALI cell clusters. The transcription factors *BLIMP1* and *KLF4* are associated with EBV lytic reactivation in differentiating keratinocytes [32]. The largest difference was observed in cluster 6 with EBV^high^ cells, where all genes were expressed significantly higher than the averaged expression across clusters (S8 Fig), consistent with the association of cellular differentiation with EBV lytic infection. However, analysis of the same genes in cluster 2 between the EBV^latentlow^ and EBV^lytic^ cells showed lower expression of almost all (except *KLF4*, *SPRR1A*) differentiation genes in the EBV^lytic^ cells. Thus, genes that mark cellular differentiation in squamous epithelia do not distinguish EBV lytic infection in pseudostratified epithelia.

The expression levels of host surface receptors known to mediate binding to viral surface proteins were analyzed for the different cell types. The B-lymphocyte receptor *CR2* was almost exclusively expressed in B-cells (cluster 4), while *CR1* was not detected in any cluster (S9A Fig). The epithelial receptor genes *EPHA2*, *ITGAV*, *ITGB5*, *ITGB6* and *ITGB8* were expressed sparsely in the B-cell cluster and cluster 4 was therefore omitted from further comparison. Cells in cluster 6, with the highest percentage of EBV-infected cells (Fig 5C), expressed higher than average levels of *EPHA2* and *ITGB8*, and lower than average levels of *ITGB5* and *ITGB6* (S9B Fig). Not surprisingly, the expression of integrins *ITGAV*, *ITGB5* and *ITGB6* was highest in the basal cell cluster (cluster 5). Additionally, all receptor genes were downregulated in the EBV^lytic^ group of cluster 2, compared with EBV^latentlow^ cells (S9C Fig). We also analyzed publicly available scRNA-seq data from non-tumor-derived nasopharyngeal samples and found no evidence of EBV infection by alignment to EBV genes [39]. Only the expression of *EPHA2*, *ITGAV* and *ITGB8* differed between the nasopharyngeal cells from some donors (S9D Fig). Interestingly, all the receptor genes analyzed were expressed at much lower levels in the different donors that showed no sign of EBV infection than the normalized expression values in any of the epithelial clusters in the susceptible pseudo-ALI culture. Thus, it is possible that variable expression of EBV surface receptors in the nasopharynx could influence susceptibility to EBV infection.

In order to identify possible restriction factors that restrict EBV lytic infection [33,44,45], we compared the expression levels of *C18orf25* (ARKL1), *IRF1*, *IRF7*, *IRF8*, *MX1* (*MxA*), *PIAS1* and *STAT1* between the different clusters. As expected, *IRF8* was almost exclusively expressed in the B-cells (cluster 4, S10A Fig). The most significant difference between the epithelial cells was observed in the suprabasal cluster 0, which had low expression of *IRF1* and *MX1* (S10B Fig). In cluster 2 the EBV^lytic^ cells had significantly lower expression in the majority of genes (*IRF1*, *MX1*, *STAT1*, *C18orf25*) compared with the EBV^latentlow^ cells (S10C Fig).

To determine if EBV-infected cells in the pseudo-ALI culture could contain proliferating cells, cell cycle markers were analyzed. *MKI67* was not highly expressed throughout the epithelial cells with notable exceptions of a few cells in cluster 2 (S11A Fig). These cells containing high levels of *MKI67* were represented in both the EBV-infected EBV^latentlow^ and EBV^lytic^ cells (S11A Fig). A more comprehensive cell cycle analysis showed that all cell cycle stages were represented in the EBV^latentlow^ group in all clusters (S11B Fig). All cell cycle stages were also represented within the EBV^latenthigh^ and EBV^lytic^ cells of cluster 2 (S11B Fig). These data indicate that EBV-infected cells in pseudo-ALI culture are cycling and that the latently-infected cells have the potential to replicate akin to the EBV-infected latent cells in NPC tumors.

Overall, the collective analysis of cellular differentiation factors, surface receptors and restriction factors are consistent with the notion that elevated expression of at least some of the surface receptors correlate with EBV susceptibility and that the reduced expression of restriction factors are associated with permissiveness to lytic infection. While all clusters in the pseudo-ALI culture showed evidence of cycling cells, it is important to note that the latently-infected cells have the potential to replicate in every cell type represented in the clusters.

## Discussion

In conclusion, we demonstrate with a pseudo-ALI cell culture model that the pseudostratified epithelial cells from the nasopharynx are susceptible to EBV infection. In support of the significance of the pseudostratified epithelium to EBV infection, a recent report has also demonstrated that the pseudo-ALI culture from the nasopharynx is susceptible to EBV infection [13]. In agreement with our findings by scRNA-seq, EBV transcripts were detected in basal (p63^+^), mucosecretory (MUC5AC^+^) and ciliated (β-IV-tubulin^+^) cells [13], but we also find that latent and lytic cells can be detected in the pseudostratified cell types with suprabasal cells being the most permissive to lytic infection. While this initial report described the disruption of epithelial integrity by EBV infection, it did not demonstrate variation in donor cell susceptibility or whether the detection of a lytic transcript yielded virus production. Here, we demonstrate that productive lytic infection can be observed but only in one susceptible donor that express transcripts and histological markers consistent with pseudostratified epithelium. We show that EBV susceptibility is consistent across experiments but also reveal that there is donor variation (Table 1). All consenting donors were recruited at the time of surgery in the sinus clinic and as such had sinus co-pathologies. To minimize discomfort, collection was done under anesthesia. For ethical reasons, cytobrush scrapings in the volume needed to generate a starter culture was conducted with living donors with reason for surgery. While we acknowledge that the donor cells presented in this study originate from donors with sinus co-pathologies, our inclusion criteria were selective and only sampled the nasopharynx of donors without evidence (or sub-clinical presentation) of nasopharyngeal complications. Although EBV infection causes pathology in the nasopharynx in the form of NPC, unfortunately limited sampling has meant that it has been difficult to identify EBV-infected cells in the nasopharynx of asymptomatic carriers [6,7], even by the more sensitive RNAScope method [46]. Ultimately, our finding that there is variable donor susceptibility in nasopharyngeal cells warrants further validation *in vivo* by sensitive detection methods and using larger sampled areas.

In the absence of a mock-infection control it was somewhat difficult to assess whether the reported abundant EBV infection in ~20–60% of the cultured cells (possibly from one donor) discerned by RNAScope detection of EBER1 or BRLF1 transcripts as punctate foci, could be an over-estimate [13]. RNAScope is a more sensitive technique than our EBV molecular diagnostics by EBER-ISH with a biotinylated probe or by immunostaining of EBV antigens, but single-molecule resolution RNAScope detection can only assign EBV infection without discriminating information on the infection program. Not surprisingly, our scRNA-seq data from the pseudo-ALI culture of donor no. 4 yielded evidence of many more infected cells (63%–91%, Fig 5C) spanning all clusters than could be estimated by our EBV molecular diagnostic stains. We caution that while the presence of an EBV transcript denotes infection, selective evaluation by any one or two transcripts is not sufficient to distinguish a biologically meaningful latent or lytic infection from an abortive infection. There is increasing evidence from using sensitive methods of transcript detection at single cell resolution that EBV infection generates a spectrum of EBV transcriptional patterns [38]. Thus, we caution that EBV-infected cells *in vivo* may be missed if sensitive methods are not used but emphasize that global transcriptome analytical methods such as scRNA-seq improve our ability to infer biologically meaningful infection.

From scRNA-seq data, we defined transcriptionally distinct cell clusters according to marker genes assigned to each cell type and show that EBV transcriptional programs differ by cell type. Results from this study would indicate that host variables other than the expression of EphA2 impact susceptibility to EBV. The expression of integrins-αV and -β8 are linked to EBV binding [47] which varied in expression between the nasopharyngeal cells from some of the donors (S9D Fig). Our results are consistent with the hypothesis that restriction factors such as *IRF1*, *MX1*, *STAT1*, *C18orf25* limit lytic infection. Given that it is hard to find an EBV-infected nasopharyngeal cell in asymptomatic carriers that show no signs of dysplasia [6,7], the pseudo-ALI culture provides a method to explore the significance of EBV molecular pathogenesis in the pseudostratified epithelium. While our findings agree with prior studies conducted in oral organotypic rafts such that EBV lytic infection is confined to suprabasal cells [12,32], we also recognize that it would be important to develop such EBV infection models in organotypic rafts for the nasopharynx, in order to simulate the stratified epithelium of the nasal mucosa. Intriguingly, the latently-infected cells from all clusters were cycling. While the stratified epithelium from the tongue and tonsils are established sites of EBV replication and explain the epithelial-derived virions in saliva [48], EBV shedding in the nasopharynx has not been definitively established. Although EBV DNA is readily detected in the saliva of asymptomatic carriers, it is not abundantly detected in nasal cavity or nasopharyngeal swabs which would sample both cells and mucosal secretions [49]. From our study, we would hypothesize that the pseudostratified epithelium is not a major site for EBV shedding but some individuals may be prone to EBV lytic infection in such cells. We conclude that latent infection can occur in nasopharynx-derived basal/mucosecretory/ciliated cell types, which may harbor a non-productive EBV reservoir, and suggest that cycling latently-infected cells in the pseudostratified epithelium could be the precursor to EBV infection in NPC tumors.

## Materials and methods

### Ethics statement

The methods were performed in accordance with relevant guidelines and regulations and approved by the University of Pittsburgh Institutional Review Board (IRB). The study received approval under IRB: STUDY19030014 and were conducted in compliance with guidelines approved for the University of Pittsburgh Sinus Fluid and Tissue Bank. All individuals involved have given written formal consent.

### Samples

Primary nasopharyngeal cell samples were collected at UPMC Mercy hospital before emergence of the COVID-19 pandemic. Voluntary informed consent was obtained for the collection, storage and analysis of biologic and/or genetic material for research, and such de-identified samples and de-identified data may be shared with other investigators for health research.

### Cell culture

The HK1 NPC cell line and the Akata Burkitt’s lymphoma B-cell line were maintained in RPMI supplemented with 10% fetal bovine serum. HK1 and Akata cells infected with the EBV recombinant Akata strain (courtesy of Dr. George Tsao, Hong Kong University) were supplemented with 800 μg/mL G418 selection [23,50]. The EBV-infected HK1 (HK1-EBV) and Akata (rAkata) cells express neomycin-resistance and EGFP from the SV40 early promoter, inserted into the EBV non-essential *BXLF1* locus, are intact for expression of the EBV miRNAs [23,51]. Cells were incubated at 37°C with 5% CO_2_ and confirmed to be negative for mycoplasma contamination by PCR. Primary nasal epithelial cells were cultured from cytobrush scrapings of the nasopharynx. Collected cells were seeded on irradiated mouse 3T3-J2 feeder fibroblasts and expanded in Georgetown media [15]. The presence of 4 μM ROCK inhibitor (Y-27632) extends the lifespan and induces the conditional reprogramming of epithelial cells [22]. Media was changed daily, and cells were sub-cultured at 1:4 seeding density. At passage 1 or 2, 1.5x10^5^ cells were seeded on human type IV placental collagen-coated transwell filters (Corning, 0.33 cm^2^, 0.4 μm, polyethylene terephthalate) in Georgetown media for 24 hours. After 24 hours apical media was removed, cultures washed once in PBS, and the basolateral media was replaced with 400 μL of ALI medium [16] supplemented with 0.5% Ultroser G Serum Substitute (PALL), denoted as UNC/USG basolateral media. Cultures were maintained at the air-liquid interface for at least 4 weeks to allow differentiation into a pseudo-ALI culture. Basolateral media was changed 3 times a week. HK1 and HK1-EBV cells were cultured at the air-liquid interface as previously described [10].

### EBV infection

rAkata EBV-infected cells was reactivated at 1x10^6^ cells/mL with a goat polyclonal anti-human IgG Fc-specific antibody (Sigma) for 48 hours. EBV-negative Akata cells were similarly treated with anti-human IgG antibody as a mock control. Virus production was confirmed by quantitative PCR for *BALF5*, as described in S1 Supplementary Methods. Reactivated Akata cells were pelleted by centrifugation and resuspended at a concentration of 1.25x10^7^ cells /mL in calcium-/magnesium-free Dulbecco’s PBS (DPBS). Primary pseudo-ALI cultures were washed in DPBS once for 5 minutes at 37°C and twice briefly at room temperature. The reactivated B-cell suspension was added to the apical surface of the pseudo-ALI culture in 200 μL, basolateral media was replaced with DPBS, and cultures were pre-incubated at 37°C for 2 hours. The basolateral DPBS was then replaced with UNC/USG media and cultures incubated for a further 48 hours at 37°C. B-cell co-culture was removed by aspiration, and pseudo-ALI cultures were washed three times in Hank’s buffered saline solution (HBSS) to remove remaining B-cells. Cultures were fixed (2 days p.i.) or incubated at 37°C for up to 5 additional days (4–7 days p.i.), changing UNC/USG basolateral media every 48 hours.

### Single cell RNA-sequencing

Cell suspensions were loaded into 10X Genomics Chromium instrument for library preparation as described previously [52], using the single cell 3’v3.1 (SC3Pv3) chemistry. Library QC was performed on an Agilent Bioanalyzer. High-throughput sequencing was performed by Novogene on a HiSeq paired-end 150 bp configuration yielding >472M reads.

### Code availability

The R script for Seurat workflow and for data visualization is available upon request.

## Supporting information

S1 Fig Validation of EBV molecular diagnostics. In-situ hybridization (EBER-ISH) and immunofluorescence staining (LMP1, Zebra, gp350) for EBV molecular markers in the HK1-EBV latent (2-D culture) and lytic reactivation (3-D ALI culture) cell culture model. Shown are confocal images from one Z-section. Positive staining is indicated in red and nuclei are counterstained with DAPI, blue. Scale bar = 40 μm.
(TIF)

S2 Fig EBV *de novo* infection in non-susceptible pseudo-ALI cultures. Shown are maximum intensity projections of confocal images on the *xy* (square) and *xz* (rectangle) planes. Nasopharyngeal cells in pseudo-ALI culture are stained for Zebra, gp350, LMP1, or EBER-ISH (red), and counterstained with DAPI (blue).
(TIF)

S3 Fig Anti-human IgG stains to control for contaminating B-cells from the inoculum. Stitched images of pseudo-ALI cultures stained for anti-human IgG (white) showing the entire membrane area, counterstained with DAPI (blue). Shown are examples of the control images for the corresponding stain (labeled in parentheses) in which positive staining for the EBV marker of interest was detected. The positive control (+) for anti-human IgG is an image of stained rAkata B-cells on a glass slide.
(TIF)

S4 Fig Comparison of EBV genes against EGFP gene count. Dot plot shows the total expression of EBV genes per cell plotted against the EGFP gene count per cell, displayed as UMI pseudocount (UMI counts per cell+1), from the pseudo-ALI culture of donor no. 4. Gray box denotes cells with low EGFP and low EBV counts, representing cells with low capture efficiency that may not have captured EBV or EGFP transcripts. Black box denotes cells with high EGFP count but no EBV count, indicative of abortive infection.
(TIF)

S5 Fig Separating the mixed cell cluster (cluster 6) by sub-clustering and EBV gene expression. Shown are the results for the EBV-infected pseudo-ALI culture of donor no. 4. (A) Assignment of cell types by expression of marker genes for subclusters 6–1, 6–2, 6–3 and 6–4. (B) Heatmaps show EBV gene expression by UMI count per cell, displayed for each subcluster. EBV^high^ cells can be distinguished from EBV^low^ cells by the total EBV counts (total EBV UMI counts per cell) shown at the bottom of each heatmap. Only the EBV-infected cells (with reads aligning to the EBV genome) are shown with the number of EBV-infected cells displayed for each subcluster indicated in parenthesis. EBV genes are color-coded into lytic: immediate-early (orange)/early (red)/late (green); latent (blue); and unassigned (purple). The scRNA-seq reads were aligned to the EBV genome fused annotation.
(EPS)

S6 Fig Assignment of EBV latent (low and high) and lytic subgroups by EBV gene count (nEBV) and % EBV transcripts per cell in the suprabasal cluster 2. Shown are EBV-infected cells as determined by the presence of at least one EBV gene UMI count. (A) Heatmap of EBV UMI counts for cells in cluster 2, with total EBV counts, EBV genes per cell, and % EBV transcripts per cell displayed on the bottom rows. Brackets indicates numbers of cells in each group. (B) Dot plot of EBV-infected cells grouped by EBV^latentlow^, EBV^latenthigh^ and EBV^lytic^ subgroups from cluster 2. Each dot represents a cell showing UMI counts for an immediately-early gene transcript (*BRLF1/BZLF1*) plotted against % EBV transcripts or the total number of EBV (nEBV) genes detected per cell, showing that EBV^latentlow^ has no reads aligning to *BRLF1/BZLF1*.
(TIF)

S7 Fig Analysis of EBV gene expression in NPC tumors. Heatmap of scRNA-seq of NPC tumors (no. NPC36, NPC46 and NPC50) from the study by Jin S. *et al*. 2020 aligned against the EBV (Akata, fused annotation) genome. These three NPC tumors were selected based on the highest viral content of aligned EBV reads (see S1 Table).
(EPS)

S8 Fig Comparison of the expression of cellular differentiation markers. (A) Violin plots show the expression of cellular differentiation markers grouped by (A) cell type-defined clusters or, (B) EBV infection status from the pseudo-ALI culture of donor no. 4. The Kruskal-Wallis non-parametric ANOVA test evaluates the expression difference across all clusters. The Wilcoxon signed rank non-parametric test compares two groups by cluster no. (A), or EBV infection status (B), using the population expression mean as the reference group in the cluster analysis. Box plot shows the mean, the inter-quartile ranges and the minimum/maximum.
(TIF)

S9 Fig Comparison of the expression of EBV receptors. Violin plots show the expression of EBV receptors grouped by (A, B) cell type-defined clusters or (C) EBV infection status from the pseudo-ALI culture of donor no. 4. (D) The expression of EBV receptors in nasopharyngeal (non-tumor-derived) tissue samples from the scRNA-seq dataset in the study by Jin S. *et al*. 2020. The Kruskal-Wallis non-parametric ANOVA test evaluates the expression difference across all groups. The Wilcoxon signed rank non-parametric test compares the expression in two groups by cluster number (A, B), EBV infection status (C), or nasopharyngeal sample no. (D) using the population expression mean as the reference group in the cluster analysis. Box plot shows the mean, the inter-quartile ranges and the minimum/maximum.
(TIF)

S10 Fig Comparison of the expression of EBV restriction factors. Violin plots show the expression of EBV restriction factors grouped by (A, B) cell type-defined clusters or (C) EBV infection status in the pseudo-ALI culture of donor no. 4. The Kruskal-Wallis non-parametric ANOVA test evaluates the expression difference across all clusters. The Wilcoxon signed rank non-parametric test compares the expression in two groups by cluster no. (A, B), or EBV infection status (C) using the population expression mean as the reference group in the cluster analysis. Box plot shows the mean, the inter-quartile ranges and the minimum/maximum.
(TIF)

S11 Fig Comparison of cell cycle markers. (A) Violin plots show the expression of MKI67 grouped by cell type-defined clusters or by EBV infection status from the pseudo-ALI culture of donor no. 4. The Kruskal-Wallis non-parametric ANOVA test evaluates the expression difference across all clusters. The Wilcoxon signed rank non-parametric test was used to compare two groups by cluster no. or EBV infection status, using the population expression mean as the reference group in the cluster analysis. (B) Ridgeline plots of EBV-infected cells grouped by EBV^latentlow^, EBV^latenthigh^ and EBV^lytic^ subgroups from cluster 2 of the pseudo-ALI culture from donor no. 4. Shown is the cell cycle score for the G1, S and G2/M phase plotted against percentage of EBV transcripts per cell. Plots in gray illustrate no cells in the displayed category.
(EPS)

S1 Table Classification of cells and EBV-quantification of scRNA-seq datasets. Single cell RNA-seq data from donor no, 4 and the study by Jin S. *et al*. 2020 were aligned against the EBV (Akata, fused annotation) genome and scored for EBV-positive and -negative cells.
(XLSX)

S2 Table Differentially-expressed genes in the EBV^latentlow^/EBV^latenthigh^/EBV^lytic^ subgroups and in nasopharyngeal tissues. EBV^latentlow^, EBV^latenthigh^ and EBV^lytic^ subgroups were compared for differentially expressed genes. A second comparison of (1) EBV-positive NPC tumors, (2) EBV-infected pseudo-ALI and (3) non-tumor nasopharyngeal epithelial cells (NPH epithelium) were compared against EBV-uninfected pseudo-ALI cells as control (ctrl). Overlapping genes in the second comparison groups are shown. The pseudo-ALI culture from donor no. 4 were compared for EBV^latentlow^ cells in cluster 2 (C2) as well as across all epithelial clusters (epi-clus).
(XLSX)

S3 Table Summary of antibodies and staining reagents. (DOCX)

S1 Supplementary Methods Supplementary materials and methods for EBV molecular analysis, scRNA-seq analysis and statistical tests. (DOCX)

## References

[1] Young LS, Yap LF, Murray PG. Epstein-Barr virus: more than 50 years old and still providing surprises. Nature reviews Cancer. 2016;16(12):789–802. Epub 2016/11/04. 10.1038/nrc.2016.92 . 27687982

[2] Raab-Traub N. Nasopharyngeal Carcinoma: An Evolving Role for the Epstein-Barr Virus. Current topics in microbiology and immunology. 2015;390:339–63. Epub 2015/10/02. 10.1007/978-3-319-22822-8_14 . 26424653

[3] Shair KHY, Reddy A, Cooper VS. New Insights from Elucidating the Role of LMP1 in Nasopharyngeal Carcinoma. Cancers [Internet]. 2018 3 21; 10(4). 10.3390/cancers10040086 29561768 PMC5923341

[4] Tsao SW, Tsang CM, Pang PS, Zhang G, Chen H, Lo KW. The biology of EBV infection in human epithelial cells. Seminars in cancer biology. 2012;22(2):137–43. Epub 2012/04/13. 10.1016/j.semcancer.2012.02.004 . 22497025

[5] Tsang CM, Deng W, Yip YL, Zeng MS, Lo KW, Tsao SW. Epstein-Barr virus infection and persistence in nasopharyngeal epithelial cells. Chin J Cancer. 2014;33(11):549–55. Epub 2014/09/17. 10.5732/cjc.014.10169 . 25223910 PMC4244318

[6] Pathmanathan R, Prasad U, Sadler R, Flynn K, Raab-Traub N. Clonal proliferations of cells infected with Epstein-Barr virus in preinvasive lesions related to nasopharyngeal carcinoma. The New England journal of medicine. 1995;333(11):693–8. 10.1056/NEJM199509143331103 . 7637746

[7] Sam CK, Brooks LA, Niedobitek G, Young LS, Prasad U, Rickinson AB. Analysis of Epstein-Barr virus infection in nasopharyngeal biopsies from a group at high risk of nasopharyngeal carcinoma. International journal of cancer Journal international du cancer. 1993;53(6):957–62. 10.1002/ijc.2910530616 . 8386141

[8] Chan AS, To KF, Lo KW, Mak KF, Pak W, Chiu B, et al. High frequency of chromosome 3p deletion in histologically normal nasopharyngeal epithelia from southern Chinese. Cancer research. 2000;60(19):5365–70. Epub 2000/10/18. . 11034072

[9] Shair KHY. mSphere of Influence: 3-D Culture Models Influence Studies on Epstein-Barr Virus Molecular Pathogenesis in the Epithelium. mSphere. 2020;5(5). Epub 2020/09/25. 10.1128/mSphere.00954-20 . 32968011 PMC7520808

[10] Caves EA, Cook SA, Lee N, Stoltz D, Watkins S, Shair KHY. Air-Liquid Interface Method To Study Epstein-Barr Virus Pathogenesis in Nasopharyngeal Epithelial Cells. mSphere. 2018;3(4). Epub 2018/07/20. 10.1128/mSphere.00152-18 . 30021875 PMC6052337

[11] Hutt-Fletcher LM. The Long and Complicated Relationship between Epstein-Barr Virus and Epithelial Cells. Journal of virology. 2017;91(1). 10.1128/JVI.01677-16 . 27795426 PMC5165189

[12] Temple RM, Zhu J, Budgeon L, Christensen ND, Meyers C, Sample CE. Efficient replication of Epstein-Barr virus in stratified epithelium in vitro. Proceedings of the National Academy of Sciences of the United States of America. 2014;111(46):16544–9. Epub 2014/10/15. 10.1073/pnas.1400818111 . 25313069 PMC4246336

[13] Yu F, Lu Y, Li Y, Uchio Y, Pangnguriseng UA, Kartika AV, et al. Epstein-Barr Virus Infection of Pseudostratified Nasopharyngeal Epithelium Disrupts Epithelial Integrity. Cancers (Basel). 2020;12(9). Epub 2020/09/26. 10.3390/cancers12092722 . 32972034 PMC7564236

[14] Ali MY. Histology of the human nasopharyngeal mucosa. J Anat. 1965;99(Pt 3):657–72. Epub 1965/07/01. . 5857093 PMC1270703

[15] Serrano Castillo F, Bertrand CA, Myerburg MM, Shapiro ME, Corcoran TE, Parker RS. A physiologically-motivated model of cystic fibrosis liquid and solute transport dynamics across primary human nasal epithelia. J Pharmacokinet Pharmacodyn. 2019;46(5):457–72. Epub 2019/09/09. 10.1007/s10928-019-09649-0 . 31494805

[16] Fulcher ML, Randell SH. Human nasal and tracheo-bronchial respiratory epithelial cell culture. Methods in molecular biology. 945. 2012/10/26 ed2013. p. 109–21. 10.1007/978-1-62703-125-7_8 23097104

[17] Chen S, Schoen J. Air-liquid interface cell culture: From airway epithelium to the female reproductive tract. Reprod Domest Anim. 2019;54 Suppl 3:38–45. Epub 2019/09/13. 10.1111/rda.13481 . 31512315

[18] Richard M, van den Brand JMA, Bestebroer TM, Lexmond P, de Meulder D, Fouchier RAM, et al. Influenza A viruses are transmitted via the air from the nasal respiratory epithelium of ferrets. Nature communications. 2020;11(1):766. Epub 2020/02/09. 10.1038/s41467-020-14626-0 . 32034144 PMC7005743

[19] Anderson CS, Chu CY, Wang Q, Mereness JA, Ren Y, Donlon K, et al. CX3CR1 as a respiratory syncytial virus receptor in pediatric human lung. Pediatr Res. 2020;87(5):862–7. Epub 2019/11/15. 10.1038/s41390-019-0677-0 . 31726465 PMC7774023

[20] Hao W, Bernard K, Patel N, Ulbrandt N, Feng H, Svabek C, et al. Infection and propagation of human rhinovirus C in human airway epithelial cells. Journal of virology. 2012;86(24):13524–32. Epub 2012/10/05. 10.1128/JVI.02094-12 . 23035218 PMC3503113

[21] Lu R, Zhao X, Li J, Niu P, Yang B, Wu H, et al. Genomic characterisation and epidemiology of 2019 novel coronavirus: implications for virus origins and receptor binding. Lancet. 2020;395(10224):565–74. Epub 2020/02/03. 10.1016/S0140-6736(20)30251-8 . 32007145 PMC7159086

[22] Liu X, Ory V, Chapman S, Yuan H, Albanese C, Kallakury B, et al. ROCK inhibitor and feeder cells induce the conditional reprogramming of epithelial cells. The American journal of pathology. 2012;180(2):599–607. Epub 2011/12/23. 10.1016/j.ajpath.2011.10.036 . 22189618 PMC3349876

[23] Maruo S, Yang L, Takada K. Roles of Epstein-Barr virus glycoproteins gp350 and gp25 in the infection of human epithelial cells. The Journal of general virology. 2001;82(Pt 10):2373–83. Epub 2001/09/20. 10.1099/0022-1317-82-10-2373 . 11562531

[24] Webster-Cyriaque J, Middeldorp J, Raab-Traub N. Hairy leukoplakia: an unusual combination of transforming and permissive Epstein-Barr virus infections. Journal of virology. 2000;74(16):7610–8. 10.1128/jvi.74.16.7610-7618.2000 . 10906215 PMC112282

[25] Gilligan K, Rajadurai P, Resnick L, Raab-Traub N. Epstein-Barr virus small nuclear RNAs are not expressed in permissively infected cells in AIDS-associated leukoplakia. Proceedings of the National Academy of Sciences of the United States of America. 1990;87(22):8790–4. 10.1073/pnas.87.22.8790 . 2174165 PMC55045

[26] Tsai ST, Jin YT, Mann RB, Ambinder RF. Epstein-Barr virus detection in nasopharyngeal tissues of patients with suspected nasopharyngeal carcinoma. Cancer. 1998;82(8):1449–53. Epub 1998/04/29. . 9554519

[27] Niedobitek G, Young LS, Lau R, Brooks L, Greenspan D, Greenspan JS, et al. Epstein-Barr virus infection in oral hairy leukoplakia: virus replication in the absence of a detectable latent phase. The Journal of general virology. 1991;72 (Pt 12):3035–46. Epub 1991/12/01. 10.1099/0022-1317-72-12-3035 . 1662695

[28] Caves EA, Butch RM, Cook SA, Wasil LR, Chen C, Di YP, et al. Latent Membrane Protein 1 Is a Novel Determinant of Epstein-Barr Virus Genome Persistence and Reactivation. mSphere. 2017;2(6). Epub 2017/11/15. 10.1128/mSphereDirect.00453-17 . 29134204 PMC5677982

[29] Chen J, Sathiyamoorthy K, Zhang X, Schaller S, Perez White BE, Jardetzky TS, et al. Ephrin receptor A2 is a functional entry receptor for Epstein-Barr virus. Nat Microbiol. 2018;3(2):172–80. 10.1038/s41564-017-0081-7 . 29292384 PMC5972547

[30] Zhang H, Li Y, Wang HB, Zhang A, Chen ML, Fang ZX, et al. Ephrin receptor A2 is an epithelial cell receptor for Epstein-Barr virus entry. Nat Microbiol. 2018;3(2):164–71. 10.1038/s41564-017-0080-8 . 29292383

[31] Merrifield J, O’Donnell R, Davies DE, Djukanovic R, Wilson SJ. A panel of antibodies for identifying squamous metaplasia in endobronchial biopsies from smokers. Biotech Histochem. 2011;86(5):340–4. Epub 2010/07/29. 10.3109/10520295.2010.502844 . 20662603

[32] Nawandar DM, Wang A, Makielski K, Lee D, Ma S, Barlow E, et al. Differentiation-Dependent KLF4 Expression Promotes Lytic Epstein-Barr Virus Infection in Epithelial Cells. PLoS pathogens. 2015;11(10):e1005195. 10.1371/journal.ppat.1005195 . 26431332 PMC4592227

[33] Liu X, Sadaoka T, Krogmann T, Cohen JI. Epstein-Barr Virus (EBV) Tegument Protein BGLF2 Suppresses Type I Interferon Signaling To Promote EBV Reactivation. Journal of virology. 2020;94(11). Epub 2020/03/28. 10.1128/JVI.00258-20 . 32213613 PMC7269453

[34] Depledge DP, Mohr I, Wilson AC. Going the Distance: Optimizing RNA-Seq Strategies for Transcriptomic Analysis of Complex Viral Genomes. Journal of virology. 2019;93(1). Epub 2018/10/12. 10.1128/JVI.01342-18 . 30305358 PMC6288342

[35] Drayman N, Patel P, Vistain L, Tay S. HSV-1 single-cell analysis reveals the activation of anti-viral and developmental programs in distinct sub-populations. Elife. 2019;8. Epub 2019/05/16. 10.7554/eLife.46339 . 31090537 PMC6570482

[36] Wyler E, Franke V, Menegatti J, Kocks C, Boltengagen A, Praktiknjo S, et al. Single-cell RNA-sequencing of herpes simplex virus 1-infected cells connects NRF2 activation to an antiviral program. Nature communications. 2019;10(1):4878. Epub 2019/10/28. 10.1038/s41467-019-12894-z . 31653857 PMC6814756

[37] Shnayder M, Nachshon A, Rozman B, Bernshtein B, Lavi M, Fein N, et al. Single cell analysis reveals human cytomegalovirus drives latently infected cells towards an anergic-like monocyte state. Elife. 2020;9. Epub 2020/01/23. 10.7554/eLife.52168 . 31967545 PMC7039680

[38] SoRelle ED, Dai J, Bonglack EN, Heckenberg EM, Zhou JY, Giamberardino SN, et al. Single-cell RNA-seq reveals transcriptomic heterogeneity mediated by host-pathogen dynamics in lymphoblastoid cell lines. Elife. 2021;10. Epub 2021/01/28. 10.7554/eLife.62586 . 33501914 PMC7867410

[39] Jin S, Li R, Chen MY, Yu C, Tang LQ, Liu YM, et al. Single-cell transcriptomic analysis defines the interplay between tumor cells, viral infection, and the microenvironment in nasopharyngeal carcinoma. Cell research. 2020;30(11):950–65. Epub 2020/09/10. 10.1038/s41422-020-00402-8 . 32901110 PMC7784966

[40] Liu Y, He S, Wang XL, Peng W, Chen QY, Chi DM, et al. Tumour heterogeneity and intercellular networks of nasopharyngeal carcinoma at single cell resolution. Nature communications. 2021;12(1):741. Epub 2021/02/04. 10.1038/s41467-021-21043-4 . 33531485 PMC7854640

[41] Ruiz Garcia S, Deprez M, Lebrigand K, Cavard A, Paquet A, Arguel MJ, et al. Novel dynamics of human mucociliary differentiation revealed by single-cell RNA sequencing of nasal epithelial cultures. Development. 2019;146(20). Epub 2019/09/29. 10.1242/dev.177428 . 31558434 PMC6826037

[42] Vieira Braga FA, Kar G, Berg M, Carpaij OA, Polanski K, Simon LM, et al. A cellular census of human lungs identifies novel cell states in health and in asthma. Nature medicine. 2019;25(7):1153–63. Epub 2019/06/19. 10.1038/s41591-019-0468-5 . 31209336

[43] Arvey A, Tempera I, Tsai K, Chen HS, Tikhmyanova N, Klichinsky M, et al. An atlas of the Epstein-Barr virus transcriptome and epigenome reveals host-virus regulatory interactions. Cell host & microbe. 2012;12(2):233–45. Epub 2012/08/21. 10.1016/j.chom.2012.06.008 . 22901543 PMC3424516

[44] Siddiqi UZ, Vaidya AS, Li X, Marcon E, Tsao SW, Greenblatt J, et al. Identification of ARKL1 as a Negative Regulator of Epstein-Barr Virus Reactivation. Journal of virology. 2019;93(20). Epub 2019/07/26. 10.1128/JVI.00989-19 . 31341047 PMC6798110

[45] Zhang K, Lv DW, Li R. Protein inhibitor of activated STAT1 (PIAS1) inhibits IRF8 activation of Epstein-Barr virus lytic gene expression. Virology. 2020;540:75–87. Epub 2019/11/20. 10.1016/j.virol.2019.11.011 . 31743858 PMC6957754

[46] Yu F, Lu Y, Petersson F, Wang DY, Loh KS. Presence of lytic Epstein-Barr virus infection in nasopharyngeal carcinoma. Head & neck. 2018;40(7):1515–23. Epub 2018/03/10. 10.1002/hed.25131 . 29522272

[47] Chesnokova LS, Nishimura SL, Hutt-Fletcher LM. Fusion of epithelial cells by Epstein-Barr virus proteins is triggered by binding of viral glycoproteins gHgL to integrins alphavbeta6 or alphavbeta8. Proceedings of the National Academy of Sciences of the United States of America. 2009;106(48):20464–9. 10.1073/pnas.0907508106 . 19920174 PMC2787161

[48] Hadinoto V, Shapiro M, Sun CC, Thorley-Lawson DA. The dynamics of EBV shedding implicate a central role for epithelial cells in amplifying viral output. PLoS pathogens. 2009;5(7):e1000496. Epub 2009/07/07. 10.1371/journal.ppat.1000496 . 19578433 PMC2698984

[49] Coghill AE, Wang CP, Verkuijilen S, Yu KJ, Hsu WL, Middeldorp JM, et al. Evaluation of nasal and nasopharyngeal swab collection for the detection of Epstein-Barr virus in nasopharyngeal carcinoma. Journal of medical virology. 2018;90(1):191–5. Epub 2017/08/24. 10.1002/jmv.24918 . 28833336

[50] Lo AK, Lo KW, Tsao SW, Wong HL, Hui JW, To KF, et al. Epstein-Barr virus infection alters cellular signal cascades in human nasopharyngeal epithelial cells. Neoplasia. 2006;8(3):173–80. 10.1593/neo.05625 . 16611410 PMC1578522

[51] Lo AK, To KF, Lo KW, Lung RW, Hui JW, Liao G, et al. Modulation of LMP1 protein expression by EBV-encoded microRNAs. Proceedings of the National Academy of Sciences of the United States of America. 2007;104(41):16164–9. 10.1073/pnas.0702896104 . 17911266 PMC2042179

[52] Morse C, Tabib T, Sembrat J, Buschur KL, Bittar HT, Valenzi E, et al. Proliferating SPP1/MERTK-expressing macrophages in idiopathic pulmonary fibrosis. Eur Respir J. 2019;54(2). Epub 2019/06/22. 10.1183/13993003.02441-2018 . 31221805 PMC8025672

