# Supplementary figures and images for: A primary nasopharyngeal three-dimensional air-liquid interface cell culture model of the pseudostratified epithelium reveals differential donor- and cell type-specific susceptibility to Epstein-Barr virus infection
Source: PLoS Pathog. 2021 Apr 29;17(4):e1009041. doi: 10.1371/journal.ppat.1009041 (PMC8112674; doi:10.1371/journal.ppat.1009041)

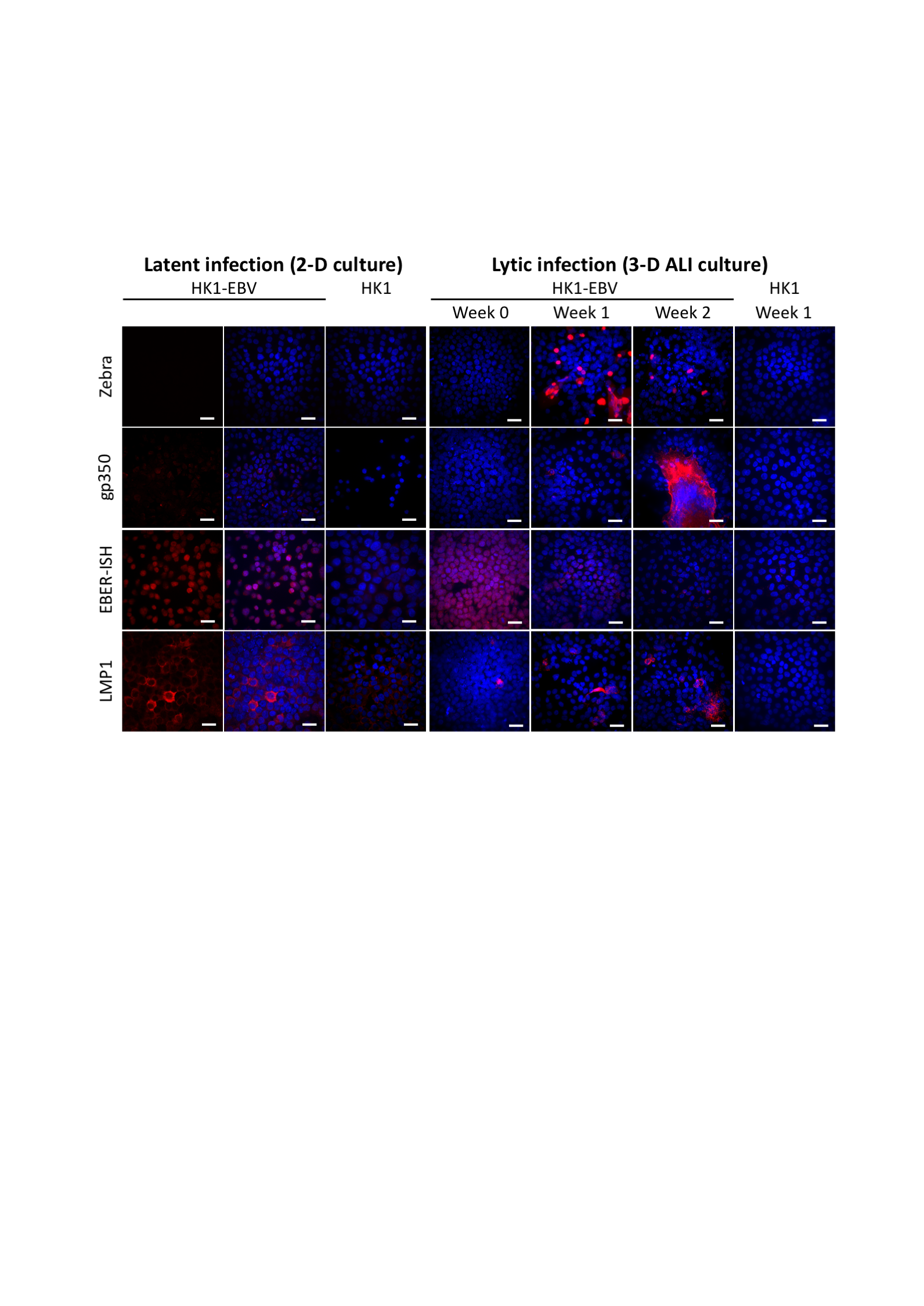

Supplement: S1 Fig — In-situ hybridization (EBER-ISH) and immunofluorescence staining (LMP1, Zebra, gp350) for EBV molecular markers in the HK1-EBV latent (2-D culture) and lytic reactivation (3-D ALI culture) cell culture model. Shown are confocal images from one Z-section. Positive staining is indicated in red and nuclei are counterstained with DAPI, blue. Scale bar = 40 μm. (TIF) [file ppat.1009041.s001.tif]

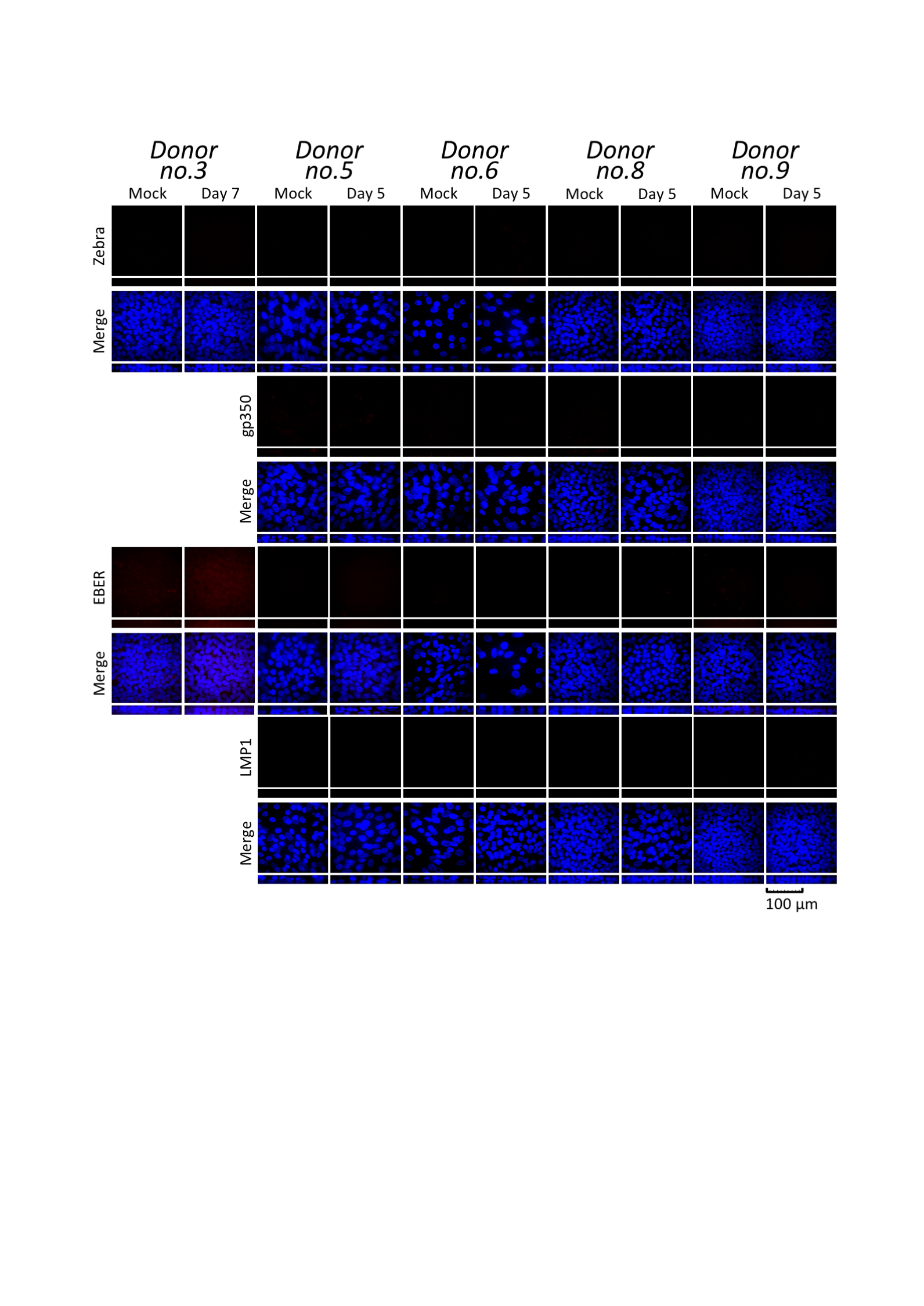

Supplement: S2 Fig — Shown are maximum intensity projections of confocal images on the xy (square) and xz (rectangle) planes. Nasopharyngeal cells in pseudo-ALI culture are stained for Zebra, gp350, LMP1, or EBER-ISH (red), and counterstained with DAPI (blue). (TIF) [file ppat.1009041.s002.tif]

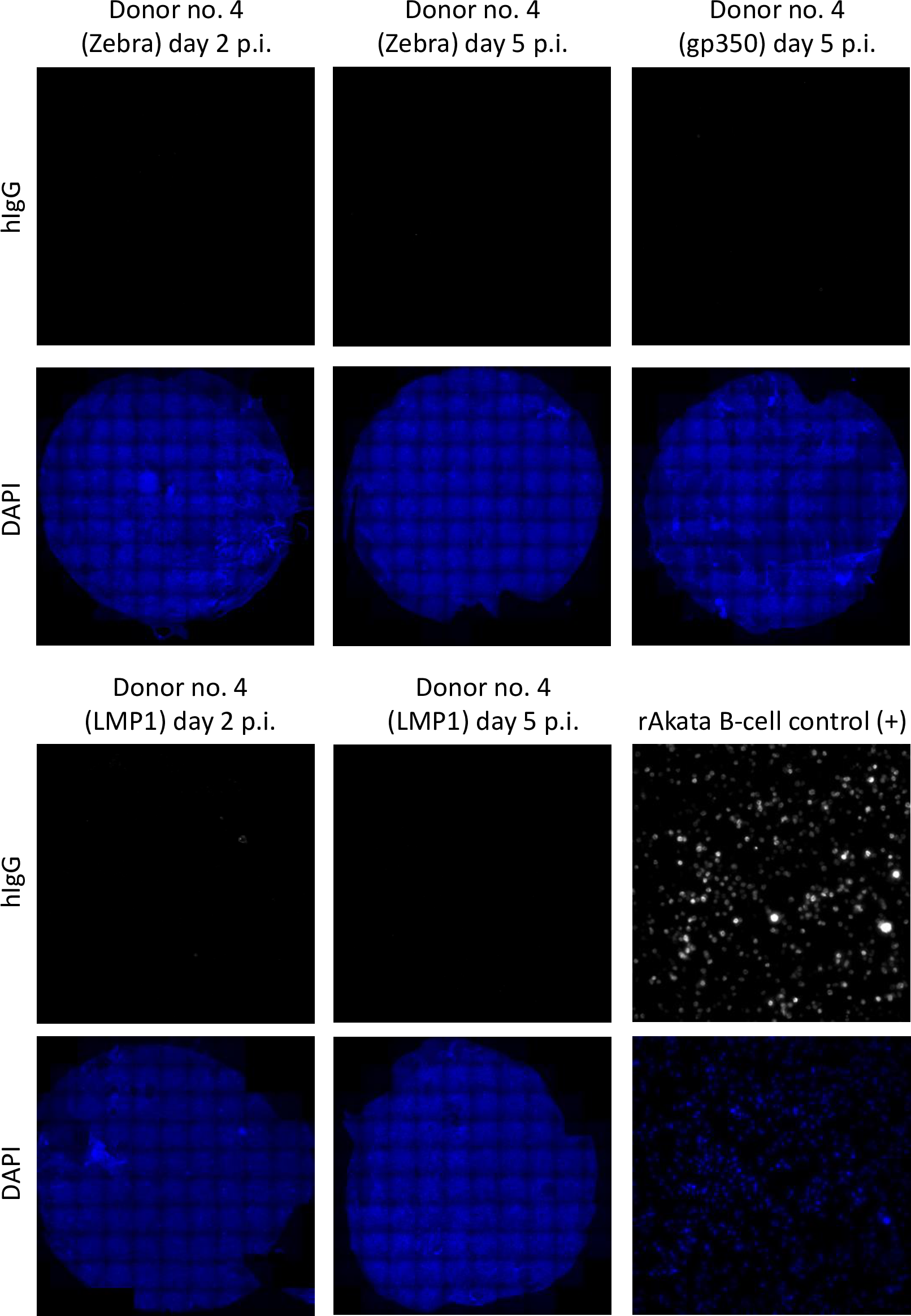

Supplement: S3 Fig — Stitched images of pseudo-ALI cultures stained for anti-human IgG (white) showing the entire membrane area, counterstained with DAPI (blue). Shown are examples of the control images for the corresponding stain (labeled in parentheses) in which positive staining for the EBV marker of interest was detected. The positive control (+) for anti-human IgG is an image of stained rAkata B-cells on a glass slide. (TIF) [file ppat.1009041.s003.tif]

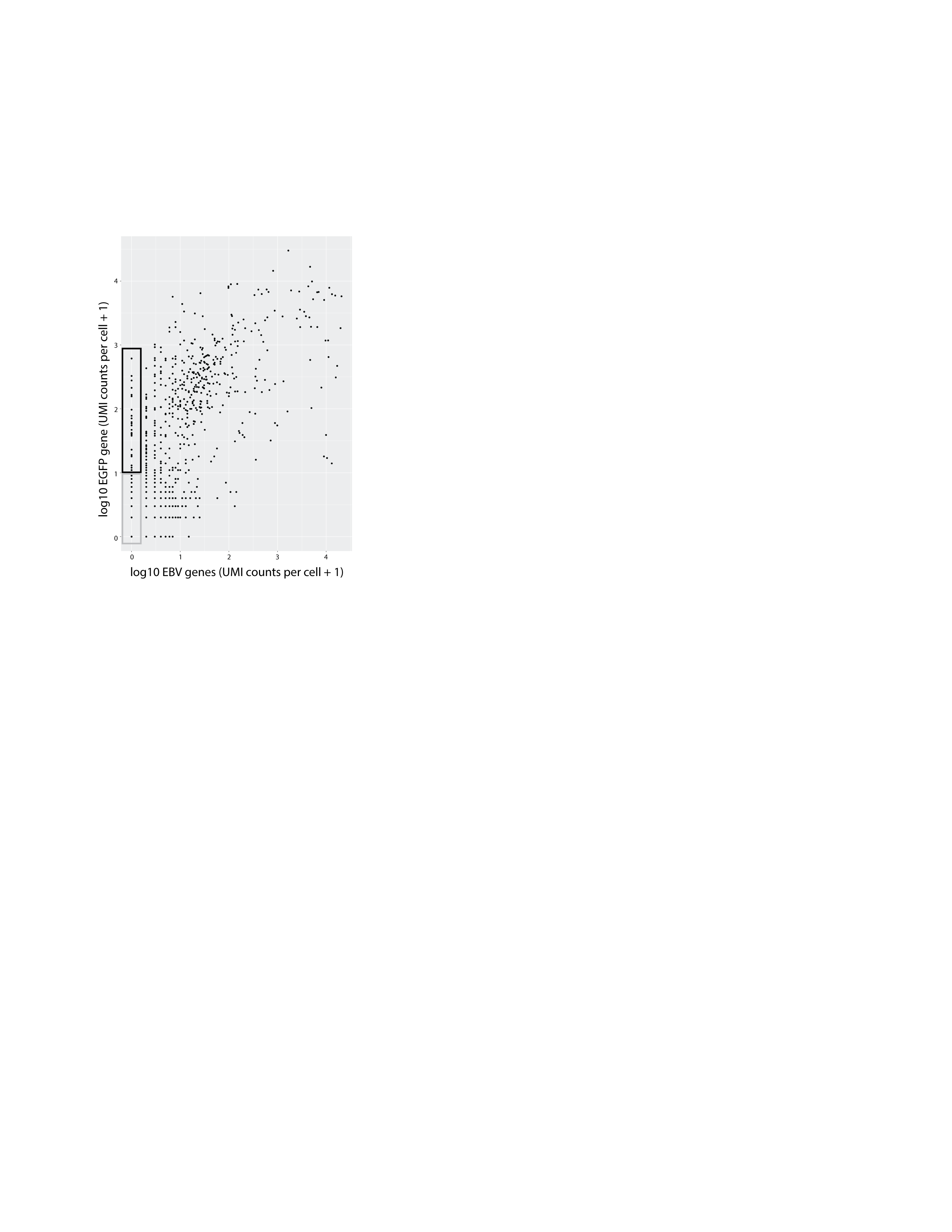

Supplement: S4 Fig — Dot plot shows the total expression of EBV genes per cell plotted against the EGFP gene count per cell, displayed as UMI pseudocount (UMI counts per cell+1), from the pseudo-ALI culture of donor no. 4. Gray box denotes cells with low EGFP and low EBV counts, representing cells with low capture efficiency that may not have captured EBV or EGFP transcripts. Black box denotes cells with high EGFP count but no EBV count, indicative of abortive infection. (TIF) [file ppat.1009041.s004.tif]

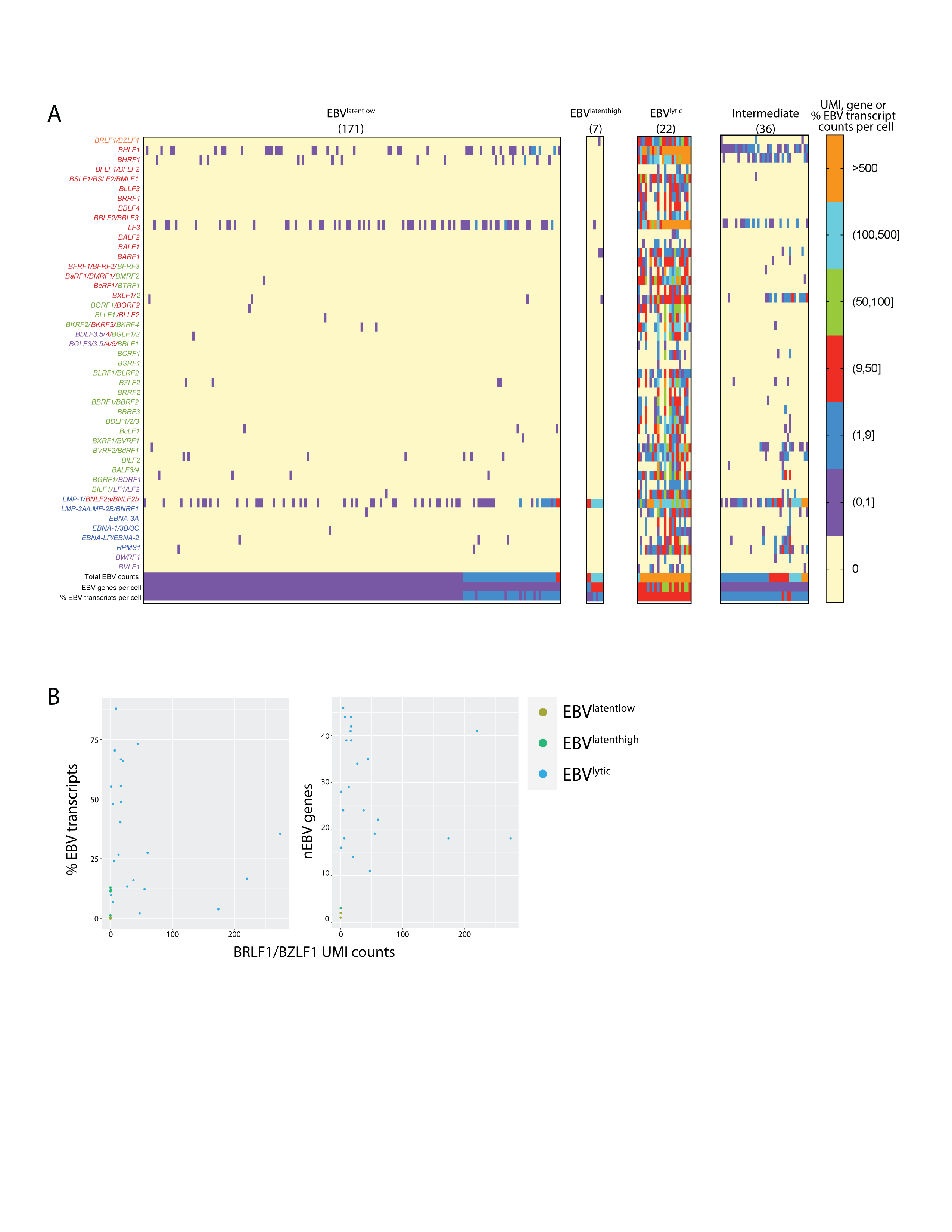

Supplement: S6 Fig — Shown are EBV-infected cells as determined by the presence of at least one EBV gene UMI count. (A) Heatmap of EBV UMI counts for cells in cluster 2, with total EBV counts, EBV genes per cell, and % EBV transcripts per cell displayed on the bottom rows. Brackets indicates numbers of cells in each group. (B) Dot plot of EBV-infected cells grouped by EBVlatentlow, EBVlatenthigh and EBVlytic subgroups from cluster 2. Each dot represents a cell showing UMI counts for an immediately-early gene transcript (BRLF1/BZLF1) plotted against % EBV transcripts or the total number of EBV (nEBV) genes detected per cell, showing that EBVlatentlow has no reads aligning to BRLF1/BZLF1. (TIF) [file ppat.1009041.s006.tif]

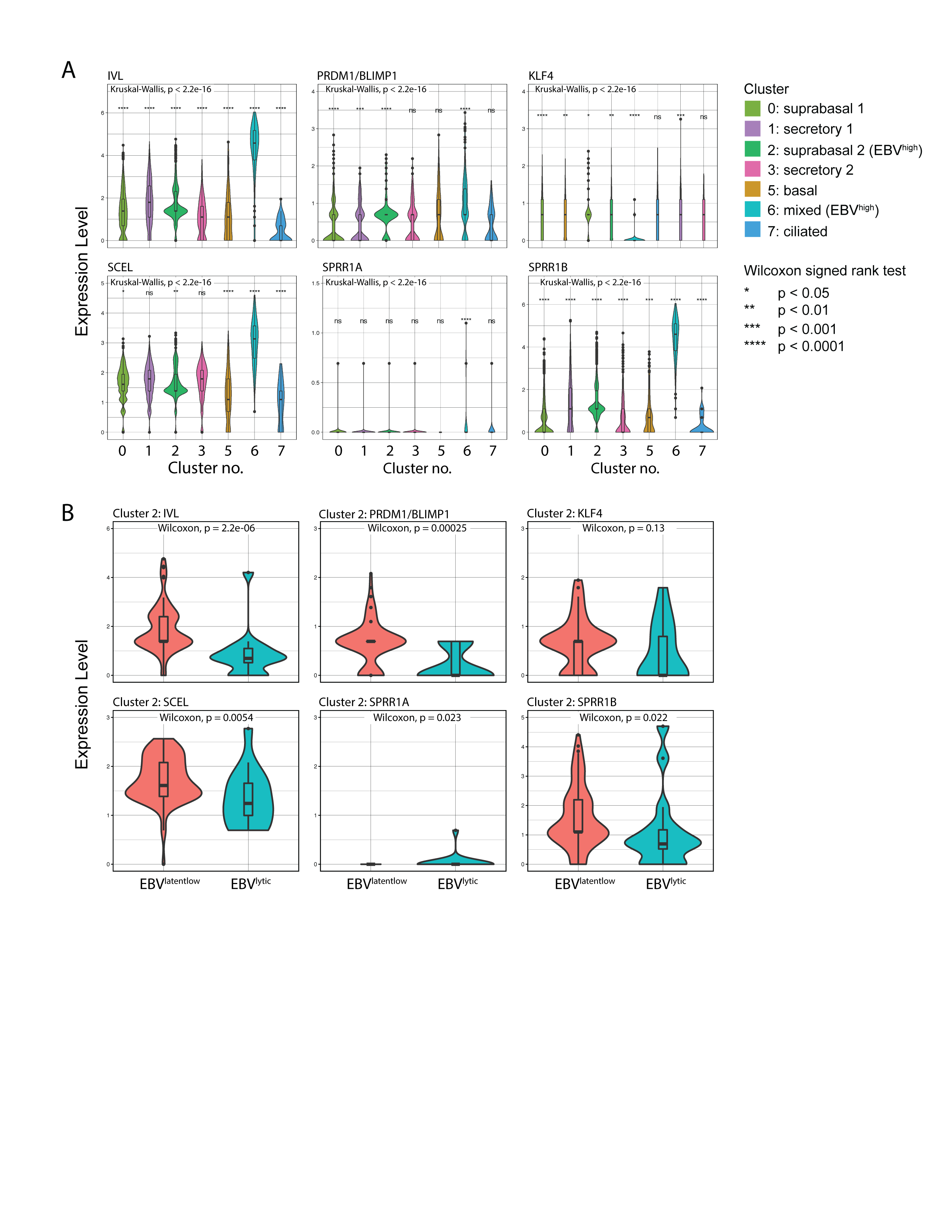

Supplement: S8 Fig — (A) Violin plots show the expression of cellular differentiation markers grouped by (A) cell type-defined clusters or, (B) EBV infection status from the pseudo-ALI culture of donor no. 4. The Kruskal-Wallis non-parametric ANOVA test evaluates the expression difference across all clusters. The Wilcoxon signed rank non-parametric test compares two groups by cluster no. (A), or EBV infection status (B), using the population expression mean as the reference group in the cluster analysis. Box plot shows the mean, the inter-quartile ranges and the minimum/maximum. (TIF) [file ppat.1009041.s008.tif]

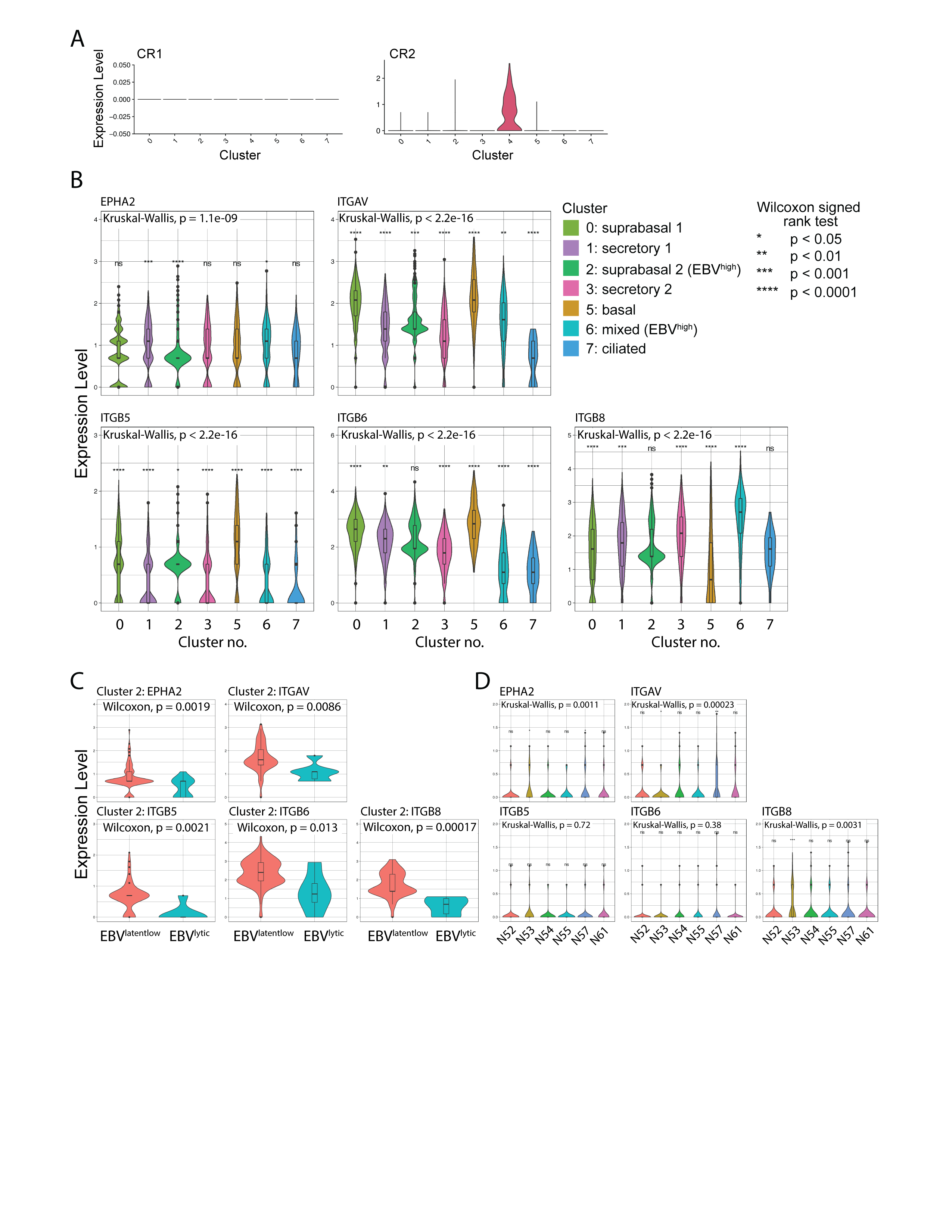

Supplement: S9 Fig — Violin plots show the expression of EBV receptors grouped by (A, B) cell type-defined clusters or (C) EBV infection status from the pseudo-ALI culture of donor no. 4. (D) The expression of EBV receptors in nasopharyngeal (non-tumor-derived) tissue samples from the scRNA-seq dataset in the study by Jin S. et al. 2020. The Kruskal-Wallis non-parametric ANOVA test evaluates the expression difference across all groups. The Wilcoxon signed rank non-parametric test compares the expression in two groups by cluster number (A, B), EBV infection status (C), or nasopharyngeal sample no. (D) using the population expression mean as the reference group in the cluster analysis. Box plot shows the mean, the inter-quartile ranges and the minimum/maximum. (TIF) [file ppat.1009041.s009.tif]

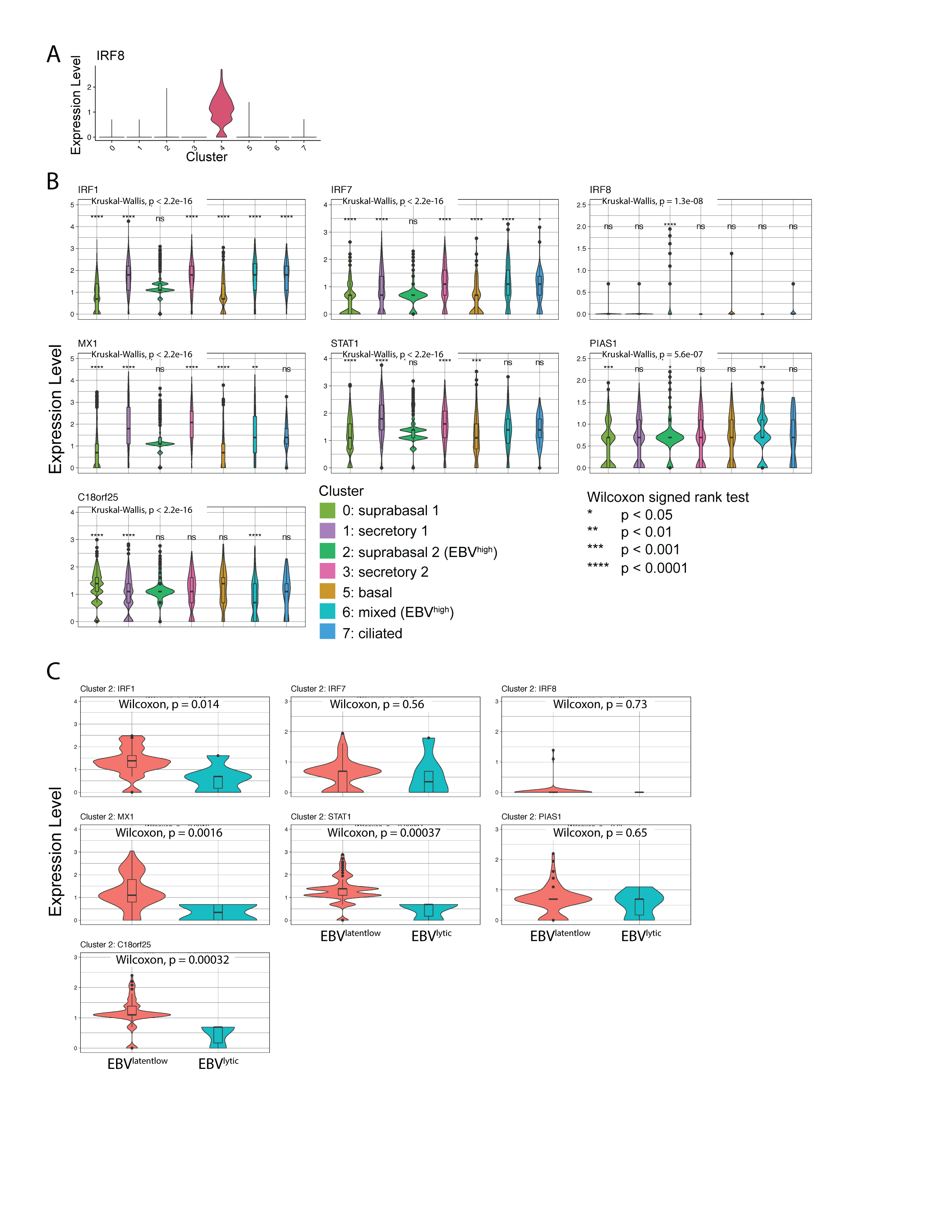

Supplement: S10 Fig — Violin plots show the expression of EBV restriction factors grouped by (A, B) cell type-defined clusters or (C) EBV infection status in the pseudo-ALI culture of donor no. 4. The Kruskal-Wallis non-parametric ANOVA test evaluates the expression difference across all clusters. The Wilcoxon signed rank non-parametric test compares the expression in two groups by cluster no. (A, B), or EBV infection status (C) using the population expression mean as the reference group in the cluster analysis. Box plot shows the mean, the inter-quartile ranges and the minimum/maximum. (TIF) [file ppat.1009041.s010.tif]
