# Supplementary material for: A primary nasopharyngeal three-dimensional air-liquid interface cell culture model of the pseudostratified epithelium reveals differential donor- and cell type-specific susceptibility to Epstein-Barr virus infection
Source: PLoS Pathog. 2021 Apr 29;17(4):e1009041. doi: 10.1371/journal.ppat.1009041 (PMC8112674; doi:10.1371/journal.ppat.1009041)
Supplement: S3 Table — (DOCX) [file ppat.1009041.s014.docx]

S3 Table. Summary of antibodies and staining reagents.

| Stain | Target | Antibody (Ab)/Probe/Reagent | Antibody/Probe/Reagent Name | Concentration | Vendor | Part Number |
| --- | --- | --- | --- | --- | --- | --- |
| 1 | Zebra | Primary Ab | Mouse anti-Zebra (clone BZ1) | 2 μg/mL | Santa Cruz Biotechnology | sc-53904 |
|  |  | Secondary Ab | Donkey anti-mouse IgG, Cy3 | 1 μg/mL | Jackson Immunoresearch | 715-165-150 |
| 2 | LMP1 | Primary Ab | Mouse anti-LMP1 (clone CS1-4) | 10 μg/mL | Abcam | ab78113 |
|  |  | Secondary Ab | Donkey anti-mouse Cy3 | 1 μg/mL | Jackson Immunoresearch | 715-165-150 |
| 3 | gp350 | Primary Ab | Mouse anti-gp350 (clone 0221) | 1 μg/mL | Santa Cruz Biotechnology | sc-57724 |
|  |  | Secondary Ab | Donkey anti-mouse IgG, Biotin-SP | 1 μg/mL | Jackson Immunoresearch | NC9673676 |
|  |  | Tertiary/Development Reagent | Donkey anti-mouse IgG, Biotin-SP | 1 μg/mL | Jackson Immunoresearch | NC9673676 |
| 4 | Human IgG | Primary Ab | Goat anti-human IgG, A647 | 2 μg/mL | Invitrogen | A-21445 |
| 5 | EphA2* | Primary Ab | Rabbit anti-EphA2 (clone D4A2) | 0.45 μg/mL | Cell Signaling Technologies | 6997S |
|  |  | Secondary Ab | SignalStain Boost Reagent (HRP, rabbit) | Undiluted | Cell Signaling Technologies | 8114S |
|  |  | Development Reagent | DAB | As recommended | Zytovision | T-1063-40 |
| 6 | EBER | EBER probe | ZytoFAST EBER Biotin Probe | Undiluted | Zytovision | T-1014-400 |
|  |  | Conjugate | Streptavidin-Rhodamine Red-X | 2 μg/mL | Jackson Immunoresearch | 016-290-084 |
| 7 | CK7* | Primary Ab | Mouse anti-cytokeratin 7 (clone OV-TL) | 1:400 | Dako | M701829-2 |
|  |  | Secondary Ab | Donkey anti-mouse Cy3 | 1 μg/mL | Jackson Immunoresearch | 715-165-150 |
| 8 | BLIMP1* | Primary Ab | Rabbit anti-BLIMP1 | 1:100 | Cell Signaling Technologies | 9115 |
|  |  | Secondary Ab | Donkey anti-rabbit IgG, AlexaFluor-488 | 2.5 μg/mL | Jackson Immunoresearch | 711546152 |
| 9 | KLF4 | Primary Ab | Rabbit anti-KLF4 | 1:100 | Cell Signaling Technologies | 4038 |
|  |  | Secondary Ab | Donkey anti-rabbit IgG, AlexaFluor-488 | 2.5 μg/mL | Jackson Immunoresearch | 711546152 |
| 10 | Inv* | Primary Ab | Mouse anti-involucrin (clone SY5) | 1:1000 | Sigma | I9018 |
|  |  | Secondary Ab | Donkey anti-mouse Cy3 | 1 μg/mL | Jackson Immunoresearch | 715-165-150 |
| 11 | K5* | Primary Ab | Rabbit anti-keratin 5 | 1:4000 | Biolegend | 905501 |
|  |  | Secondary Ab | Donkey anti-rabbit IgG, AlexaFluor-488 | 2.5 μg/mL | Jackson Immunoresearch | 711546152 |
| 12 | α-tubulin* | Primary Ab | Mouse anti-α-tubulin | 1:250 | Santa Cruz Biotechnology | sc-32293 |
|  |  | Secondary Ab | Donkey anti-mouse Cy3 | 1 μg/mL | Jackson Immunoresearch | 715-165-150 |
| 13 | MUC5AC* | Primary Ab | Mouse anti-MUC5AC (clone 45M1) | 1:1000 | Thermo Scientific | Ma1-38223 |
|  |  | Secondary Ab | Donkey anti-mouse Cy3 | 1 μg/mL | Jackson Immunoresearch | 715-165-150 |

*Heat-induced antigen retrieval (HIER) method: EDTA pH8.0, 15 mins
