# Supplementary material for: A primary nasopharyngeal three-dimensional air-liquid interface cell culture model of the pseudostratified epithelium reveals differential donor- and cell type-specific susceptibility to Epstein-Barr virus infection
Source: PLoS Pathog. 2021 Apr 29;17(4):e1009041. doi: 10.1371/journal.ppat.1009041 (PMC8112674; doi:10.1371/journal.ppat.1009041)
Supplement: S1 Supplementary Methods — (DOCX) [file ppat.1009041.s015.docx]

**SUPPLEMENTARY METHODS**

**Analysis of virus production**. Pseudo-ALI cultures were harvested 2 days p.i. (immediately after removal of B-cells and 3 HBSS washes) or 5 days p.i. by scraping in 100 μL total DPBS. Cells were pelleted by centrifugation at 500 xg for 5 min. Supernatant was removed and collected as extracellular virus. Cells were lysed by repeated freeze thawing, pelleted by centrifugation at >20000 xg for 10 min, and supernatant collected as cell-associated virus. To degrade unencapsidated cell-free DNA, supernatant was treated with 2 units of Turbo DNase (Thermo Scientific) per 50 μL supernatant for 30 mins at 37^o^C followed by inactivation for 10 mins at 70^o^C, and 100 μg/mL proteinase K (Fisher Scientific) for 30 mins at 50^o^C to release encapsidated DNA followed by inactivation at 75^o^C for 20 mins. EBV genomes were quantified by amplifying a region of *BALF5* using 0.3 μM each of primers with the sequences: 5′ GAGCGATCTTGGCAATCTCT 3′ and 5′ TGGTCATGGATCTGCTAAACC 3′. Quantitative PCR reactions were assembled using the Maxima SYBR Green qPCR master mix kit (Thermo Scientific) as per the manufacturer’s instructions, with 2 μL of supernatant containing template DNA, ROX as a passive reference dye, and absolute quantitation against a standard curve. Reactions were performed on the QuantStudio 3 instrument (Applied Biosystems) and analyzed with QuantStudio Design and Analysis Software (v1.4.1). Mean values and standard deviation were calculated from technical triplicates. A standard curve was generated using 25 to 10^6^ copies of a plasmid containing a fragment of *BALF5*. Harvested supernatant was titrated by the Green Raji Unit (GRU) method as previously described [1].

**Immunofluorescence, immunohistochemistry and image analysis.** Immunostaining of EBV proteins was performed on whole mount transwell membranes as previously described [1]. EBV-encoded RNAs (EBERs) were detected by fluorescent in-situ hybridization as per the manufacturer’s protocol (Zytovision). Detection and post hybridization steps were performed according to the immunofluorescence protein staining protocol. Primary antibodies, concentrations, and detection reagents are listed in S3 Table. Confocal images were captured on an Olympus IX81 inverted microscope, CREST X-Light V2 spinning disc confocal, with a 20x (numerical aperture [NA] = 0.45), 60x oil (NA = 1.45), or 100x oil (NA = 1.4) objective, and a Hamamatsu Flash 4.0 LT camera. Maximum intensity projections of z-stack images of ALI cultures (z spacing = 170 nm) were generated using Olympus CellSens Dimension software. Histograms comparing intensity of staining for EBV markers were generated by combining the 580 nm channel of the mock image and infected image in CellSens Dimension. LMP1 particle analysis was performed using Fiji software (https://imagej.net/Fiji). Particle intensity, circularity and size thresholds were applied to the red fluorescent channel. The Fiji plugin, analyze particles, was used to determine intensity of particles with parameters of 0-1 circularity and 0-0.001 pixel^2^ size. For histology, whole transwell membranes were fixed overnight in 4% paraformaldehyde. Paraffin embedding, Hematoxylin & eosin (H&E), Alcian blue/periodic acid Schiff, pan-cytokeratin (Dako, clone AE1/AE3), and Ki67 (Dako, clone MIB-1) staining were performed by the University of Pittsburgh Research Histology Services. Immunohistochemistry for Ephrin receptor A2 (Cell Signaling Technology, clone D4A2) was performed as per the manufacturer’s protocol with the following modification: ZytoVision 3,3′-Diaminobenzidine (DAB) reagent was used for development and nuclei were counterstained with ZytoVision Nuclear Blue. Antigen retrieval and antibody staining conditions for all FFPE sections are listed in S3 Table. Brightfield images were captured on an Olympus PROVIS AX70 microscope with a QImaging QIClick camera and QCapture Pro 7 software.

**scRNA-seq analysis.** The 10X Genomics Cell Ranger (version 3.0.2) pipeline for scRNA-seq counted unique molecular identifiers (UMIs) that were confidently mapped to one exonic locus of an annotated gene in a strand-specific manner [2]. Unannotated, non-exonic reads and reads that mapped to loci of overlapping genes on the same strand were not counted. Sequencing reads were aligned to a merged human and EBV (Akata strain) genome (hg38+EBV) compiled from NCBI GenBank GRCh38.90 and KC207813.1 using the 10X Genomics Cell Ranger 3.0.2 workflow with the default parameters. The Akata EBV genome annotation was modified by combining overlapping genes forming a fused annotation. The EGFP sequence was added to the merged reference genome. Exon coordinates were added to the EBV genome using available information from annotated CDS, and when unavailable (*BSLF1, BBLF2/BBLF3, BALF3*) gene coordinates were used. Sequence coverage containing downstream complementary polyadenylation signal for *BHLF1* and *LMP-1/BNLF2a/BNLF2b* was observed and the annotations for the genes were extended accordingly. Seventy-five EBV gene annotations were added to the original 13. The gff3 files were converted to gtf using gffread from cufflinks/2.2.1. An index of the merged genomes was created with cellranger/3.0.2 mkref. The Fastq files were analyzed with cellranger/3.0.2 count applying the SC3Pv3 chemistry. More than 470M reads were captured (sequencing saturation similar across clusters) with 123,249 mean reads per cell and 71.8% reads mapped confidently to the (EBV+hg38) transcriptome. Seurat (version 3.1.4), an R package developed for single cell analysis, was used for data analysis, normalization of gene expression and visualization of cell populations [3]. Cells were clustered using shared nearest neighbor modularity by the K-Nearest Neighbor algorithm implemented in Seurat. Cells were filtered for the following inclusion criteria: total number of detected genes > 500, percentage of mitochondrial genes < 25%, percentage of hemoglobin genes < 0.025%, percentage of ribosomal genes < 40%, and total number of read counts > 1000. The epithelial cell subtypes were annotated based on defined marker genes.

**EBV status and cell cycle analysis of pseudo-ALI cells.** The EBV-expressing cells of cluster 2 were used to set the thresholds for the different EBV-stages. The cells were divided into four categories: i) EBV^lytic^, cells that expressed ten or more EBV genes and the EBV transcripts represented equal or more than 1% of the individual cell’s total UMI, ii) EBV^latenthigh^, cells that expressed less than ten EBV genes and the EBV transcripts represented equal or more than 1% of the individual cells total UMI, iii) EBV^latentlow^, cells having one or two EBV genes detected and the EBV transcripts represent less than 1% of the individual cells total UMI, iv) Intermediate, cells that have detectable levels of EBV (and/or EGFP) but fail to fall under any of the other three categories. The UMI counts of BRLF1/BZLF1 of all cells in cluster 2 was plotted against the amount of EBV transcripts and EBV percentage to visualize the spread of the EBV^latenthigh^, EBV^latentlow^ and EBV^lytic^ groups using ggolot2 [4]. Cell cycle scores were calculated using the CellCycleScoring function in Seurat3.2.2 according to G2M and S phase gene lists with modifications [5].

**Analysis of primary NPC.** Publicly available scRNA-seq datasets of primary NPC specimens and non-malignant nasopharyngeal biopsies were analyzed [6]. Among all the NPC patient samples, EBV was detected in 13 samples, the three samples with highest EBV content (counts per million, cpm) were chosen for further analysis.

**Merging and reanalysis of datasets.** For the merging of datasets, several Seurat functions were used in a pipeline-like manner for the fusion and normalization of different samples into one cohesive dataset. Filtered and normalized samples were corrected using SC-Transform with three variables used as point of regression, nCount_RNA, percent mitochondrial transcripts and percent ribosomal transcripts. After correction, the samples were merged using the Seurat function Merge and once again normalized and corrected using SC-Transform to normalize the values so that the merged dataset were interpreted as a single dataset.

**Differential Gene Expression analysis.** The differentially expressed genes were calculated between three datasets using publicly available scRNA-seq from a previous study by García, SR., et al. [7]. The sample was referred to as HAEC_BEGM_ALI22 and acted as the reference group for all three datasets. The three differential gene expression comparisons were: i) Shair (dataset from current study): EBV^latentlow^ from cluster 2 or all epithelial clusters of pseudo-ALI from donor no. 4 vs. Reference Group, ii) MuSheng [6]: Merged NPC vs. Reference Group and iii) MuSheng [6]: Merged Healthy Control vs. Reference Group. The Seurat function FindAllMarkers was used to single out the most differential expressed genes for each dataset using a basic filtering threshold a minimum Log(FoldChange) of 0.25, a minimum percentage representation within at least one of the two compared groups of 0.25 and a minimum statistical p-value of < 0.001. The statistical p-value was calculated by the FindAllMarkers function itself. The genes that passed this filtering step were saved for further analysis in a table form, for each comparison respectively. An aggregated list was generated from these tables and additional filtering was performed in order to remove a bias based on the chosen reference group. The conditional requirement for being labeled a true differentially expressed gene was to a) Appear in both comparison i) and ii) but not in iii), as iii) serves as the control to only measure the differentially expressed genes based solely on the expression of the Reference Group and not to a cancer sample. The genes that passed this conditional requirement totaled 16 high-confident genes.

**Statistical Tests for the Violin plots.** The violin plots were generated using the R package ggplot2. The statistical tests on the violin plots were generated using the R package ggpubr. The Kruskal-Wallis one-way analysis of variance, or Kruskal-Wallis test, was generated using standard parameters and assuming independent variables. The Wilcoxon signed-rank test was generated using standard parameters with the exception of alternating the reference group dependent on the categorization. For the EBV-status specific categorization, no reference group was chosen. For the clustering specific categorization, the base-mean was used as a reference group.

**SUPPLEMENTARY REFERENCES**

1. Caves EA, Cook SA, Lee N, Stoltz D, Watkins S, Shair KHY. Air-Liquid Interface Method To Study Epstein-Barr Virus Pathogenesis in Nasopharyngeal Epithelial Cells. mSphere. 2018;3(4). Epub 2018/07/20. doi: 10.1128/mSphere.00152-18. PubMed PMID: 30021875; PubMed Central PMCID: PMCPMC6052337.

2. Zheng GX, Terry JM, Belgrader P, Ryvkin P, Bent ZW, Wilson R, et al. Massively parallel digital transcriptional profiling of single cells. Nature communications. 2017;8:14049. Epub 2017/01/17. doi: 10.1038/ncomms14049. PubMed PMID: 28091601; PubMed Central PMCID: PMCPMC5241818 L.M., D.A.M., S.Y.N., M.S.L., P.W.W., C.M.H., R.B., A.W., K.D.N., T.S.M. and B.J.H. are employees of 10x Genomics.

3. Satija R, Farrell JA, Gennert D, Schier AF, Regev A. Spatial reconstruction of single-cell gene expression data. Nature biotechnology. 2015;33(5):495-502. Epub 2015/04/14. doi: 10.1038/nbt.3192. PubMed PMID: 25867923; PubMed Central PMCID: PMCPMC4430369.

4. Wickham H. ggplot2 : Elegant Graphics for Data Analysis. Cham: Springer International Publishing : Imprint: Springer,; 2016.

5. Nestorowa S, Hamey FK, Pijuan Sala B, Diamanti E, Shepherd M, Laurenti E, et al. A single-cell resolution map of mouse hematopoietic stem and progenitor cell differentiation. Blood. 2016;128(8):e20-31. Epub 2016/07/02. doi: 10.1182/blood-2016-05-716480. PubMed PMID: 27365425; PubMed Central PMCID: PMCPMC5305050.

6. Jin S, Li R, Chen MY, Yu C, Tang LQ, Liu YM, et al. Single-cell transcriptomic analysis defines the interplay between tumor cells, viral infection, and the microenvironment in nasopharyngeal carcinoma. Cell research. 2020;30(11):950-65. Epub 2020/09/10. doi: 10.1038/s41422-020-00402-8. PubMed PMID: 32901110; PubMed Central PMCID: PMCPMC7784966.

7. Ruiz Garcia S, Deprez M, Lebrigand K, Cavard A, Paquet A, Arguel MJ, et al. Novel dynamics of human mucociliary differentiation revealed by single-cell RNA sequencing of nasal epithelial cultures. Development. 2019;146(20). Epub 2019/09/29. doi: 10.1242/dev.177428. PubMed PMID: 31558434; PubMed Central PMCID: PMCPMC6826037.
